# The Effect of Cell Growth Phase on the Regulatory Cross-Talk between Flagellar and Spi1 Virulence Gene Expression

**DOI:** 10.1371/journal.ppat.1003987

**Published:** 2014-03-06

**Authors:** Chakib Mouslim, Kelly T. Hughes

**Affiliations:** Department of Biology, University of Utah, Salt Lake City, Utah, United States of America; Osaka University, Japan

## Abstract

The flagellar regulon controls *Salmonella* biofilm formation, virulence gene expression and the production of the major surface antigen present on the cell surface: flagellin. At the top of a flagellar regulatory hierarchy is the master operon, *flhDC*, which encodes the FlhD_4_C_2_ transcriptional complex required for the expression of flagellar, chemotaxis and *Salmonella* pathogenicity island 1 (Spi1) genes. Of six potential transcriptional start-sites within the *flhDC* promoter region, only two, P1*_flhDC_* and P5*_flhDC_*, were functional in a wild-type background, while P6*_flhDC_* was functional in the absence of CRP. These promoters are transcribed differentially to control either flagellar or Spi1 virulent gene expression at different stages of cell growth. Transcription from P1*_flhDC_* initiates flagellar assembly and a negative autoregulatory loop through FlhD_4_C_2_-dependent transcription of the *rflM* gene, which encodes a repressor of *flhDC* transcription. Transcription from P1*_flhDC_* also initiates transcription of the Spi1 regulatory gene, *hilD*, whose product, in addition to activating Spi1 genes, also activates transcription of the *flhDC* P5 promoter later in the cell growth phase. The regulators of *flhDC* transcription (RcsB, LrhA, RflM, HilD, SlyA and RtsB) also exert their control at different stages of the cell growth phase and are also subjected to cell growth phase control. This dynamic of *flhDC* transcription separates the roles of FlhD_4_C_2_ transcriptional activation into an early cell growth phase role for flagellar production from a late cell growth phase role in virulence gene expression.

## Author Summary

Flagellar-mediated motility is fundamental to *Salmonella* pathogenesis, which takes the lives of hundreds of thousands of people each year. The genes of the *Salmonella* pathogenicity island 1 and those of the flagellar regulon are part of the same transcriptional hierarchy. We report the novel finding where the key control of this network takes place at the *flhDC* promoter region. We followed the transcription from the two “live” *flhDC* promoters as a function of the cell growth phase. P1 comes on early in the cell cycle, while P5 comes on late. Transcription of P5 is HilD dependent, which represents a totally new finding and *Salmonella* specific: there is no HilD in *E. coli flhDC* control, no P5 transcription. P1 & P5 can express *flhDC* to equivalent levels, yet only P1- dependent expression produces motility UNLESS we artificially induce P5 EARLY in the cell cycle. This work is the foundation for the cell cycle stages a *Salmonella* bacterium experiences during host infection. This is a significant conceptual advance in *Salmonella* pathogenesis: one can no longer consider gene regulation at 37°C and OD 0.6 as a reflection of the *Salmonella* infection cycle; the whole cell growth cycle must be considered in understanding this complex biological processes.

## Introduction

Tens of millions of human cases of Salmonellosis, a foodborne gastroenteritis caused by *Salmonella enterica*, occur worldwide every year killing more than a hundred thousand people annually (World Health Organization Fact sheet N°139, August 2013). Typhoid fever caused by *Salmonella* Typhi kills an equivalent number of people each year. A prominent player in *Salmonella* pathogenesis is the bacterial flagellum. The bacterial flagellum is an ion-powered, complex motor organelle that endows bacterial cells, such as *Escherichia coli* and *Salmonella enterica*, with the ability to propel themselves through liquid medium and across hydrated surfaces [1]. Motility also plays an important role in biofilm formation and in the ability of many pathogens to reach their sites of infection and establish disease [2], [3].

Early work on the discovery of *Salmonella* virulence genes identified a transposon insertion in the flagellar filament cap gene, *fliD*, as defective for survival of cells in macrophages [4]. However, *fliD* is in an operon with the *fliT* gene whose product is a regulator of the flagellar and Spi1 virulence genes master regulatory complex FlhD_4_C_2_ [5], [6]. The transposon insertion in *fliD* was polar on *fliT* gene expression and thus identified regulation of FlhD_4_C_2_ activity as critical for *Salmonella* virulence. The two proteins that make up the FlhD_4_C_2_ transcriptional regulatory complex are co-expressed from the *flhDC* operon, class 1 promoter, which is at the top of a complex transcriptional hierarchy for both flagellar and Spi1 virulence genes expression. The decision whether or not to produce flagella is regulated at the levels of *flhDC* transcription, translation, FlhD_4_C_2_ assembly and stability [7]. Positive regulators of *flhDC* operon transcription include cAMP-CRP, Fis, Fur, H-NS and QseB [8]–[14]. A large number of regulatory factors are also reported to inhibit *flhDC* transcription. These factors include, LrhA, RcsB, RtsB, SlyA, DskA, PefI-SrgD, FimZ, HdfR, OmpR and RflM [15]–[20]. The FlhD_4_C_2_ activity generates an auto-regulatory loop by activating transcription of the *rflM* gene encoding a LysR-type DNA binding protein RflM, which in turn inhibits the transcription of *flhDC* [21]. The post-transcriptional factors regulating *flhDC* include, CsrA [22], [23], Hsp70 chaperone DnaK [24] and ClpXP protease [25]. Recently an FlhD_4_C_2_ repressed gene, *ydiV* [26], was shown to code for a protein (YdiV) that will bind to FlhD_4_C_2_, in its free or DNA-bound form, remove FlhD_4_C_2_ from DNA and serves as an adapter that targets FlhD_4_C_2_ for ClpXP-dependent degradation [27], [28].

In *Salmonella*, an initial characterization of the *flhDC* promoter region identified six transcriptional start sites (TSSs) [13]. In a recent study, only four of the original six TSSs were detected [29]. The presence of six TSSs in the *Salmonella flhDC* regulatory region combined with the presence of DNA binding sites of CRP, LrhA, RtsB, HilD, RcsB, HNS and others indicated a complex level of the *flhDC* transcriptional regulation.

*Salmonella enterica* is an intracellular facultative pathogen causing a range of diseases in a variety of hosts [30]. Important virulence factors required for *Salmonella* invasion of epithelial cells and development of Salmonellosis are encoded within the *Salmonella* pathogenicity island 1 (Spi1) genes. Spi1 encodes a virulence-associated type III secretion system (T3SS) as part of an injectisome structure required for the secretion and injection of multiple effector proteins into the cytoplasm of host cells [31]–[36]. Expression of Spi1 genes is controlled in response to specific combinations of environmental signals in a complex hierarchical process with multiple transcriptional regulators. These include, HilA, a member of the OmpR/ToxR family of transcriptional regulators, which promotes transcription of genes encoding the necessary components for a functional Spi1 injectisome system [32], [35], [37], [38]. Also included are the *hilC* and *hilD* genes whose products are members of the Ara/XylS family of transcriptional regulators that control *hilA* gene transcription. HilD is at the top of the regulatory network controlling Spi1 expression because most regulators controlling *hilA* transcription appears to be HilD-dependent [39], [40].

It is noteworthy to mention that many protein components of the Spi1 and flagella T3SS exhibit a significant degree of amino acid identity, leading to the production of remarkably similar T3SS structures [16], [33], [34], [41], [42]. Furthermore, many of the transcriptional and posttranslational regulatory factors of *flhDC* also target the main transcriptional regulators of Spi1, such as HilA and HilD [11], [43]–[52]. In addition, the ATP-dependent Lon protease was shown to degrade both FlhD_4_C_2_ and HilD [24], [25]. Coordinated expression of Spi1 and flagellar genes has been recently demonstrated [53]. In *Salmonella*, expression of Spi1 genes is activated by FliZ [54]–[57], which is encoded within the flagellar *fliAZY* operon. FilZ activates the *hilD* gene expression at the posttranslational level and HilD in turn promotes transcription of the *rtsAB* operon, which encodes a pathogenesis-related DNA-binding regulatory proteins. RtsA and RtsB reciprocally regulate both the Spi1 and flagellar genes [17]. The direct binding of RtsB to the *flhDC* promoter region inhibits *flhDC* transcription and motility [17].

We decided to investigate how input from different regulatory factors might integrate multiple environmental or cell cycle signals into the control of *flhDC* expression in *Salmonella enterica*. We explored how and when positive and negative regulators affect *flhDC* expression throughout the cell growth cycle. We measured the effect of RcsB, LrhA, RflM, SlyA, RtsB and HilD regulatory factors on *flhDC* operon transcription at different cell growth phases. We characterized the specific TSSs within the *flhDC* promoter region and their involvement in the positive and negative control of *flhDC* cell-cycle dependent transcription. Finally, we examined how the individual TSSs and protein regulatory factors controlled the interconnection between the flagellar and Spi1 regulons.

## Results

### Dynamics of *flhDC* operon transcription in liquid culture after induction from stationary phase

**Figure 1:**
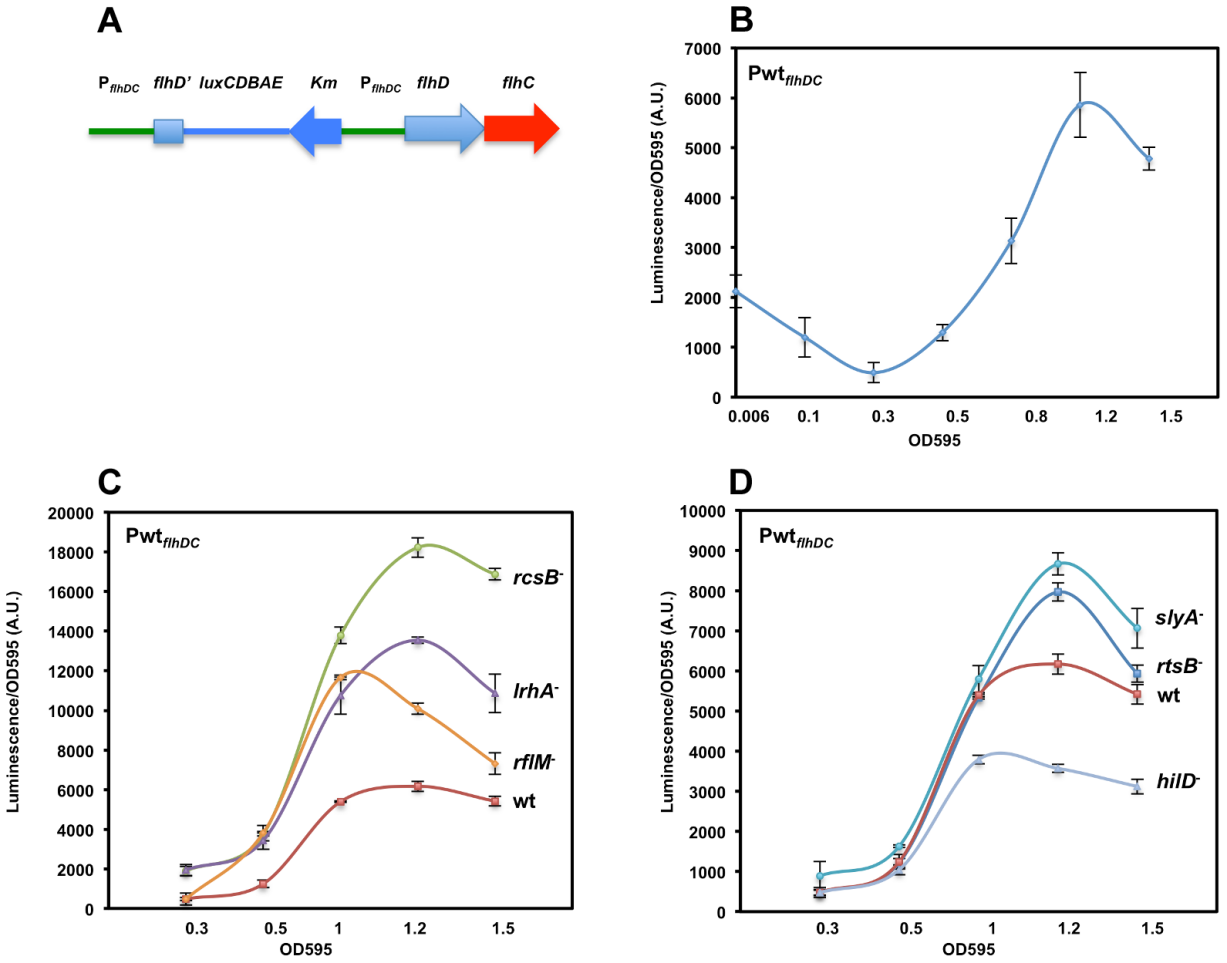
Growth phase dependent transcription of the *flhDC* operon promoter in *Salmonella enterica* serovar Typhimurium is controlled by LrhA, RcsB, RflM, HilD, SlyA and RtsB. (**A**) Diagram depicting a duplicated chromosomal region that includes fusion of the *flhDC* promoter region (P*_flhDC_*, a 728 bp upstream of the start codon of *flhD* and the first 272 nucleotides of *flhD* coding region) to the luciferase operon of *Photorhabdus luminescens* in addition to a wild-type *flhDC* promoter-operon region. (**B**) A time course plot showing P*_flhDC_*-*lux* expression at increasing cell density of strain Pwt*_flhDC_-luxCDBAE-*Pwt*_flhDC_flhD^+^C^+^* (TH18684) grown in LB media at 30°C with shaking. Luciferase activity was measured along with the OD595. Plots represent the recorded luciferase activity divided by the OD595. (**C & D**) Time course plots showing Pwt*_flhDC_*-*lux* expression at increasing cell density in the absence of *flhDC* regulators. Individual regulators of *flhDC* promoter (Pwt*_flhDC_*) transcription were removed by deletion in the Pwt*_flhDC_-luxCDBAE-*Pwt*_flhDC_flhD^+^C^+^* background. Plots for specific individual strains are identified at the right of their corresponding plots (wt = wild-type (TH18684), *rcsB^−^* = Δ*rcsB*::*tetRA* (TH19230), *lrhA^−^* = Δ*lrhA*::*tetRA* (TH18722), *rflM^−^* = Δ*rflM*::FCF (TH18716), *rtsB^−^* = *rtsB*::T-POP (TH18724), *slyA^−^* = *slyA*::T-POP (TH18720) and *hilD^−^* = Δ*hilD*::*tetRA* (TH19654)). (**C**) Loss of RcsB, LrhA or RflM resulted in increased transcription of the *flhDC* operon at early growth phase. (**D**) Effect of removal of virulence-related genes *slyA*, *rtsB* or *hilD* differentially affected *flhDC* operon transcription. Deletion of either the *rtsB* or *slyA* gene resulted in increased *flhDC* operon transcription once cells reach stationary phase contrary to a deletion in the *hilD* gene, which resulted in increased *flhDC* transcription once bacterial cells enter mid exponential phase. The OD595 values are shown at the bottom of the chart. Values are the average of three independent experiments done in duplicate. Error bars represent standard deviation.

To investigate *flhDC* operon transcription at different phases of the cell growth, we constructed a transcriptional fusion of the *flhDC* promoter region to the luciferase operon of *Photorhabdus luminescence* (*luxCDBAE* operon). Because the *flhDC* operon is autoregulated negatively by RflM and positively by HilD, we designed strains harboring an intact copy of the *flhDC* operon under the control of its native promoter (P*_flhDC_*) and an in-frame fusion of a second copy of the promoter region of *flhDC* (through the first 272 nucleotides of *flhD* coding sequence) to the luciferase operon: DUP[(Pwt*_flhDC_*-*luxCDBAE*)*Km*(Pwt*_flhDC_*-*flhD^+^C* ^+^)] (Figure 1A). Thus, individual P*_flhDC_* promoter regions transcribe both the luminescence operon reporter and the *flhDC* operon. This results in a strain with luminescence readout for the level of transcriptional activation of *flhDC* under conditions that also preserves the wild-type expression of the flagellar regulon including *flhDC* autoregulation through FlhD_4_C_2_-dependent expression of *rflM* and *hilD* genes. For simplicity, we will refer to the DUP[(Pwt*_flhDC_*-*luxCDBAE*)*Km*(Pwt*_flhDC_*-*flhD^+^C* ^+^)] construct as Pwt*_flhDC_*.

Following batch inoculation of an overnight culture of the Pwt*_flhDC_* strain into fresh media with shaking at 30°C, transcription of the *flhDC* genes declined 4-fold during the initial lag phase transition to log phase growth to a minimal value (Figure 1B). This observation is consistent with that reported in an earlier study [11]. After the transition to log phase growth, transcription of *flhDC* increased more than 10-fold between OD 0.3 and 1.2, followed by a decline in *flhDC* transcription as cells enter late log and stationary phase growth (Figure 1B).

### Dynamics of *flhDC* operon transcription during cell cycle growth in liquid culture in the absence of transcriptional regulators

In *Salmonella enterica*, flagellar regulon transcription is highest during the exponential phase of growth and decays in late stationary phase [58]. Transcription of the flagellar master regulatory operon, *flhDC*, is under both negative and positive control by multiple regulatory factors. Null mutations in any one of the *rcsB*, *rflM*, *lrhA*, *slyA*, and *rtsB* genes result in increased transcription of the *flhDC* operon, which is consistent with an inhibitory activity on *flhDC* expression. HilD is an activator of *flhDC* transcription such that over-expression of the *hilD* gene increases *flhDC* expression (Singer et al. submitted). The diversity of transcription factors controlling expression of *flhDC* reflects the complexity of *flhDC* transcriptional regulation and suggests that *flhDC* transcription is controlled when *Salmonella* cells are experiencing different metabolic or environmental states, or different growth conditions under which these transcriptional factors are active. We examined both the timing and magnitude of individual regulatory proteins on *flhDC* transcriptional control throughout the cell's growth phase. We tested *flhDC* transcriptional levels as a function of the cell's growth phase in strains missing the individual negative regulators RcsB, LrhA, RflM, RtsB, SlyA and the positive regulator HilD (Figures 1C & D). As was presented above for the wild-type strain, this was done by growing Pwt*_flhDC_* cells in liquid culture at 30°C using luciferase as the reporter for *flhDC* transcription levels. Luciferase levels were determined at specific optical densities shown in Figure 1. As expected, removal of individual inhibitors resulted in an increase in *flhDC* transcription levels while removal of HilD decreased *flhDC* transcription. Importantly, our assay revealed a growth phase-dependent hierarchy of the effect of these regulators. At OD 0.3, basal *flhDC* transcription was elevated in the absence of LrhA and RcsB, while removal of RflM, RtsB, SlyA or HilD exhibited the same basal level of transcription as wild type (Figures 1C & 1D). This suggests that RcsB and LrhA act earlier, during lag phase, to inhibit *flhDC* transcription. This effect could also represent a carry-over of repression from stationary phase that keep *flhDC* transcription low during the transition to log growth. In the absence of RflM we observed an earlier transition to *flhDC* activation than in the other mutant strains. Since FlhD_4_C_2_ transcribes the *rflM* gene and RflM protein inhibits *flhDC* transcription (*flhDC* auto-inhibition), this result suggests that *flhDC* auto-inhibition through RflM occurs during early exponential phase to control when full FlhD_4_C_2_-dependent gene expression occurs at log phase. The negative effect of RtsB and SlyA on *flhDC* transcription was detected as cells enter early stationary phase. We also observed that the maximum *flhDC* transcription level peaked earlier for both the *hilD* and *rflM* mutants at OD 1, while the wild type and mutants in *rcsB*, *lrhA*, *slyA* and *rtsB* peaked around OD 1.2.

The data presented in Figure 1C demonstrate that initial *flhDC* transcription is kept low by a combination of repressors including at least RcsB and LrhA. Initial FlhD_4_C_2_ expression during the stationary to log phase transition produces enough RflM to maintain a low level of *flhDC* transcription until an OD of ∼0.3 is reached. After OD 0.3, *flhDC* transcription increased significantly, but RflM, RcsB and LrhA reduce the overall level. Interestingly, the wild-type level is balanced by the presence of the HilD activator of *flhDC* transcription, the *hilD*-activated inhibitor of *flhDC* transcription RtsB and by the virulence associated factor SlyA (Figure 1D).

### The effect of growth conditions on *flhDC* transcription as a function of cell growth

**Figure 2:**
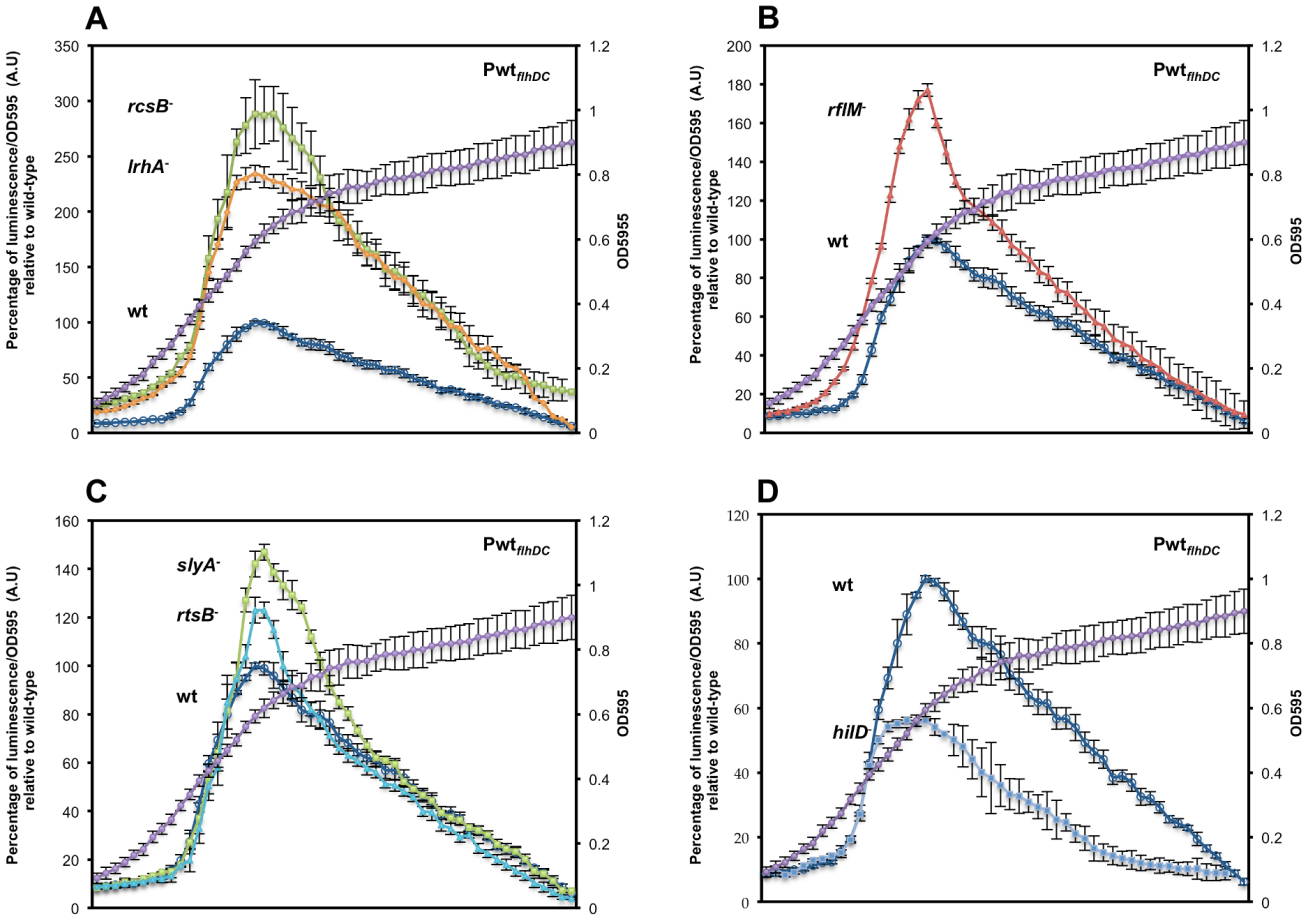
Precise transcriptional regulation of the *flhDC* operon is growth phase dependent. Transcription kinetics for the *flhDC* operon in various mutant backgrounds with the Pwt*_flhDC_-luxCDBAE*-Pwt*_flhDC_flhD^+^C^+^* reporter construct measured in a 96 well plate growth format. The luciferase activity was investigated in seven genetic backgrounds: (**A**) wild-type (TH18684) empty circles, Δ*rcsB*::*tetRA* (TH19230) filled squares, Δ*lrhA*::*tetRA* (TH18722) filled diamonds, (**B**) **Δ** *rflM*::FCF (TH18716) filled triangle (**C**) *rtsB*::T-POP (TH18724) filled circles, *slyA*::T-POP (TH18720) filled squares and (**D**) **Δ** *hilD*::*tetRA* (TH19654) filled diamonds. The genotypes of the strains are indicated in the left of their plots at the level of their maximum A.U's. Cells from overnight cultures were diluted 1 to 500 in LB and 200 µl was inoculated into 96 well dark plates that were sealed with a breath easy membrane and incubated at 30°C in a plate reader with 5 min orbital shaking at 150 rpm. After a pause of 5 second following shaking, luminescence and OD595 of the inoculated wells were read during 95 second. The luminescence was recorded with a 0.1 s integration time for normalization. Arbitrary units (A.U.) were calculated as luminescence reading divided by OD595. The average at each time point was normalized to the maximum A.U. of the wild-type strain. Each data point represents six experiments performed in triplicate in different days. Error-bars indicate standard deviations. A representative growth curve is shown in the second axis of the plots.

In order to obtain more detailed information relating the effect of specific regulatory proteins on *flhDC* transcription as a function of the cell's growth phase, we determined luciferase levels for the Pwt*_flhDC_* grown in liquid culture at 30°C in 96 well plates with a microplate reader. Using this assay system, we could measure the activity of *flhDC* transcription at shorter times intervals (6 min) with continuous shaking at 150 rpm. We observed the same trend of regulation of the *flhDC* operon as seen in batch cultures for *lrhA*, *rcsB* (Figure 2A), *rflM* (Figure 2B), *slyA* and *rtsB* (Figure 2C), and *hilD* mutants (Figure 2D). However, the pattern observed in 96 well plates was somewhat different compared to the batch growth. We observed that activation of *flhDC* transcription took place earlier at OD∼0.2 rather than OD∼0.3. Consistent with this observation, the differences between the activity of *flhDC* in wild-type versus mutant strains also occurred at an earlier OD measurement in microtiter plate growth compared to growth in batch culture. The cells in 96 well plates reached maximum expression at OD∼0.6 compared to OD∼1.2 in the batch culture. We attribute these differences to the mode of growth in 96 well plates (150 rpm) where bacterial cells are grown in much lower volumes and likely to be subjected to different oxygen levels in the medium compared to batch cultures. It has been shown that activation of *flgA*, a gene under the control of *flhDC*, under static conditions (no shaking of 96 well plates) occurred immediately after dilution of an overnight culture into LB-1% Salt [53]. When we tested the activation of *flhDC* operon in standing batch culture in LB, we observed that *flhDC* transcription increased at OD∼0.12 (Figure S1), which is earlier compared to what we observed either in batch shaking (OD∼0.3) or 96 well grown cultures (OD∼0.2). Moreover, the shutdown of *flhDC* transcription observed in standing cultures took place after cells reach an OD∼0.6 compared to shaking batch culture where the shutdown started at an OD∼1.2.

### Growth phase transcriptional dynamics of *flhDC* transcriptional regulators

Because *flhDC* transcription is differentially regulated by different transcription factors in a growth phase dependent manner, we hypothesized that the effect of each of these regulators is observed at the time when they are produced during the cell growth cycle. To investigate this possibility we placed the *luxCDBAE* operon reporter under control of the promoters of the six regulatory genes *lrhA*, *rcsB*, *rflM*, *slyA*, *rtsB* and *hilD*, whose products have been demonstrated to bind directly to the *flhDC* promoter region and monitored their expression profile at different optical densities (binding of RflM or SlyA to the *flhDC* promoter region has not been reported). We monitored the activities of these constructs in 96 well plates over time. We observed that the transcription of the autoregulated gene *lrhA* is immediately activated following dilution from an overnight culture, and before the activation of *flhDC* (Figure S2.A). Transcription of *rcsD* (which is the first gene of the *rcsDB* operon transcribed from the *rcsD* promoter) also initiated before *flhDC* (Figure S2.B), whereas transcription of *rflM* overlapped with that of *flhDC* (Figure S2.B). Since *rflM* transcription is dependent on FlhD_4_C_2_, these results suggest that low basal levels of FlhD_4_C_2_ are sufficient to promote *rflM* gene transcription. In addition, transcription of *rflM* reached a maximum at OD∼0.35 and decayed very quickly (Figure S2.B) compared to the rest of the regulators tested in this study. The transcription of *hilD* gene is under positive autoregulatory control by HilD itself [59] and by HilD-activated RtsA [17]. In addition, the product of an *flhDC* activated gene, FliZ controls HilD at a posttranslational level [54], [57]. We observed that transcription of *hilD* increased at OD of ∼0.4 (Figure S2.C), at the same time when HilD promoted transcription of *flhDC* (Figure 2D). The expression of the HilD-activated *rtsA* gene (the first gene of the *rtsAB* operon) appeared to be activated at the same time as *hilD* (Figure S2.C). Transcription of the *slyA* gene was activated just after *flhDC* transcription started and before initiation of *hilD* and *rtsA* transcription, with a peak of expression at entry into stationary phase (Figure S2.D). These results suggest that there is a hierarchy of transcription of the factors regulating *flhDC* transcription that mirrors their effect on the transcriptional regulation of the *flhDC* operon.

**Figure 3:**
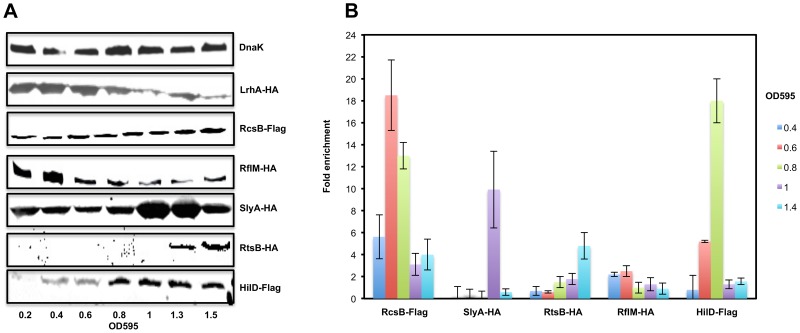
The expression levels and the in-vivo binding of regulatory factors controlling *flhDC* operon transcription during cell growth phases. (**A**) Expression of the RcsB, LrhA, RflM, SlyA, HilD and RtsB proteins in LB during growth after dilution from overnight culture. Immunoblots of whole-cell lysates of *S. typhimurium* strains carrying one of the following: a FLAG-tag in either the *rcsB* (TH18628) or *hilD* (TH20451) gene, an HA-tag in *rflM* (TH19853), *slyA* (TH19855), or *rtsB* (TH19854) gene. Growth of an individual tagged strain was monitored at OD595 and total proteins were extracted. Blotting was performed using a monoclonal anti-HA or anti-Flag antibody. Equivalent amounts of proteins (50 µg per lane) were analyzed at each time point. As a loading control, DnaK was detected using a monoclonal antibody against DnaK. The OD595 are indicated at the bottom of the figure. (**B**) In vivo binding of regulatory factors controlling *flhDC* transcription to the promoter region of *flhDC*. Chart represents the fold enrichment of the *flhDC* regulatory region DNA bound by different transcriptional factors at different ODs. Cells were grown at 30°C until they reach the ODs shown in the left of the chart. Pull-down of the DNA-protein complexes and RT-PCRs were conducted as described in Material and Methods. Fold enrichment was calculated relative to a no-antibody control as described in Material and Methods. Bars represent the average of two independent experiments of a Chromatin Immuno-precipitation assay (CHIP).

We next asked if the protein levels of the regulatory factors controlling *flhDC* transcription were also growth phase dependent. We performed Western blot analysis of whole cell lysates of *Salmonella* strains (LrhA-HA, RcsB-3×Flag, RflM-HA, SlyA-HA, RtsB-HA and HilD-Flag) at different optical densities (Figure 3A). We established that LrhA is present at an early time point during cell growth (OD∼0.2) with maximum expression at OD∼0.6 followed by a decay at late stationary phase (note that both the N-terminal and C-terminal HA-tag fusion to LrhA are made but not functional and therefore there is no positive feedback regulation of *lrhA* transcription by LrhA protein [18]). The level of RcsB protein, the transcriptional regulator of the phosphorelay system RcsDBC, also appeared to be growth phase dependent because RcsB protein was detected early in the growth phase (OD∼0.2) and increased at the stationary phase of cell growth. The FlhD_4_C_2_ activated RflM, was produced early in the growth phase (OD∼0.2), and increased at OD∼0.4 followed by a quick decay during the rest of the cell's growth phase. HilD protein, the positive activator of *flhDC* transcription, was detected at OD∼0.4 and increased at stationary phase (Figure 3A). RtsB, whose gene is under the transcriptional control by HilD, was not detected early in the growth phase and was present at OD∼1.3. The absence of RtsB at an early time point in the blot might be due to the detection limits for low protein levels in our experiment (See CHIP, Figure 3B, where RtsB was already associated with the promoter of *flhDC* at OD∼1). In contrast, the negative regulator SlyA was produced during all the phases of cell growth, with a sharp increase at OD∼1. These results demonstrate a hierarchy at the level of expression of *flhDC* regulators that specifically mimics the differential dynamics of *flhDC* operon transcription.

### *In vivo* binding by regulators of *flhDC* transcription to the *flhDC* promoter region

We examined the *in vivo* binding dynamics within the *flhDC* promoter region by these regulatory factors. At different optical densities (0.4 to 1.4), chromatin immunoprecipitations (ChIP) were conducted using strains with individually tagged transcriptional factors, RcsB, RflM, HilD, RtsB, LrhA and SlyA (Figure 3B). Expression of RcsB and binding of RscB to its target DNA at the *flhDC* promoter was detected throughout the entire growth phase. However, RcsB bound levels increased as cells progressed to exponential phase (OD 0.4 to 0.6) followed by decreased binding at latter stages of growth. The transcriptional regulator RflM binding to DNA was detected at OD∼0.4 with maximal binding at OD∼0.6, but was no longer bound the *flhDC* promoter region beyond OD∼0.6. HilD, a transcriptional activator of *flhDC*, was bound to the *flhDC* promoter region at OD∼0.4 increasing to a maximum bound level at OD∼0.8 and followed by absence of bound HilD at OD∼1. SlyA was not physically associated with the *flhDC* promoter at OD∼0.4 and ∼0.6, but was bound to the *flhDC* promoter region at OD∼0.8. There was no binding of RtsB to the *flhDC* promoter at an early time point of cell growth OD∼0.4 to 0.6. Binding by RtsB had initiated by OD 0.8 and increased through OD 1.4. We were unable to immunoprecipitate LrhA tagged protein because C-terminal or N-terminal tagged LrhA behaved like *lrhA* null mutant (Figure S5; ). These results highlight the binding dynamics of different regulators to the *flhDC* promoter region resulting in a dynamic of *flhDC* operon transcription.

### Molecular analysis of the individual *flhDC* transcriptional start-sites

**Figure 4:**
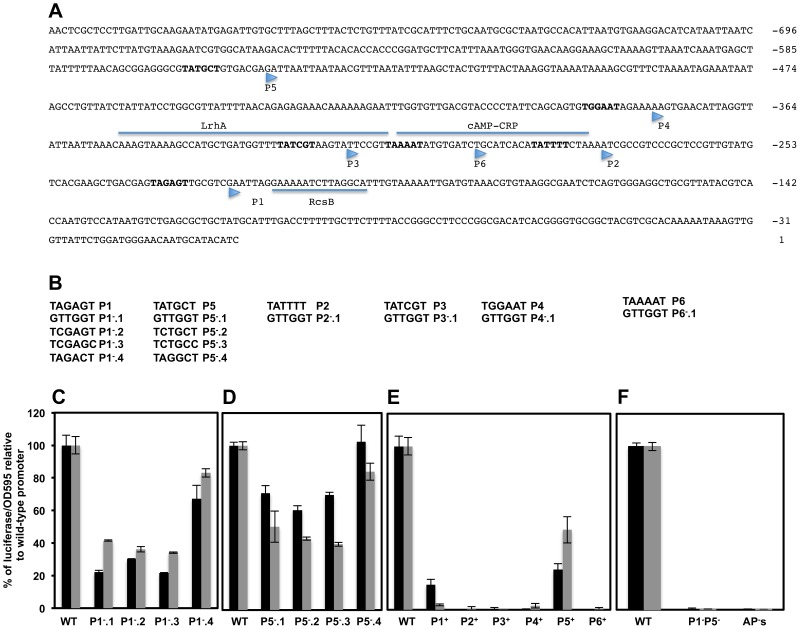
Effects of mutations in putative transcriptional start-sites within the *flhDC* promoter region on *flhDC* operon transcription. (**A**) DNA sequence and regulatory elements of the upstream regulatory region of S. *typhimurium*. Nucleotides are labeled respective to the start of the open reading frame of FlhD. The −10 box of the putative promoters are in bold and their respective transcription start site are indicated by arrowheads as determined by primer extension [13]. The transcriptional factors LrhA, RcsB, RtsB and CRP have been shown to bind directly to the *flhDC* promoter regulatory region. Experimental evidence and mutations analysis have been performed to delineate the exact binding of LrhA, RcsB and CRP (underlined). The exact binding of the RtsB has not been defined but it has been shown that RtsB binds directly to *flhDC* promoter region corresponding to a DNA fragment covering from +4 to +104 nucleotides respective to the P1 transcription start site [17]. The direct and exact binding site for SlyA, RflM and HilD transcriptional factors have not been defined yet. (**B**) DNA sequences of the −10 boxes [13] of putative transcriptional start-sites (shown as P1^−^, P2^−^, P3^−^, P4^−^, P5^−^ and P6^−^) and mutant constructs that were made in each of these −10 boxes. The individual transcriptional start-sites promoter mutants were made separately at each single −10 box or were combined together leaving only one functional −10 box out of the six described promoter start-sites (shown as P1^+^, P2^+^, P3^+^, P4^+^, P5^+^ and P6^+^). Charts represent the luciferase activities of the Pwt*_flhDC_-luxCDBAE*-Pwt*_flhDC_flhD^+^C^+^* reporter construct in wild-type and isogenic strains carrying mutations in individual start-site −10 boxes. Cells were grown overnight in LB and diluted 1 to 500 in fresh media, and grown at 30°C with shaking and luciferase activities were recorded at two optical densities (0.5, black bars and 1, grey bars). Charts of luciferase activity in strains with mutations in the P1 (**C**) and P5 (**D**) promoters of *flhDC* operon compared to the wild-type *flhDC* promoter activity that was set at 100%. Each specific mutation is indicated under their corresponding bars. (**E**) Graph of luciferase activity in strains harboring only one single wild-type −10 box of the indicated putative promoter. P1^+^ represents a strain that has only a functional P1 promoter while the rest of the promoters are mutated, etc (**F**) Luciferase activity of a strain with mutations in both P1 and P5 (P1^−^P5^−^), compared to wild type promoter and to a construct with mutations in all six promoters: AP's. Results are the average of three independent experiments done in duplicate. Error bars represent standard deviation.

Six transcriptional start sites, designated P1*_flhDC_*, P2*_flhDC_*, P3*_flhDC_*, P4*_flhDC_*, P5*_flhDC_* and P6*_flhDC_*, within *Salmonella flhDC* promoter region were obtained by primer extension [13]. However only P1*_flhDC_*, P3*_flhDC_*, P4*_flhDC_* and P5*_flhDC_* were detected by RNA-Seq based approach [29]. Each of these TSSs was preceded by a hexamer motif (−10 box) with the consensus invariant residues adenine at position 2 (A2) and thymine at position 6 (T6), except for P4 (Figure 4A). To investigate the authenticity of these TSSs, we made alterations of the −10 sequences targeting the conserved residues A2 and T6 by changing them to a cytosine residue (C) and also by totally changing the −10 box to a GTTGGT sequence (Figure 4B). As controls, additional mutations were made in each −10 box, in a nucleotide other than A2 or T6 (Figures 4B & S3A) that supposedly should not alter significantly the effect of RNAP on transcription [60]. Because *flhDC* is subjected to negative and positive transcriptional feedback, mutations of the promoters responsible for transcription of *flhDC* operon in the wild-type strain might affect the positive and negative auto-regulation of *flhDC* transcription. We thus monitored the activities of the promoters mutants fused to luciferase operon in an *flhD* ^+^ *C* ^+^ background (described above). Mutations of wild-type sequence P1*_flhDC_* (TATAGT) to GTTGGT (P1^−^.1*_flhDC_*); TCTAGC (P1^−^.2*_flhDC_*) or TCTAGT (P1^−^.3*_flhDC_*) but not TACAGT (P1^−^.4*_flhDC_*) were associated with a significant reduction of *flhDC* transcription (Figure 4C). Mutations of the wild-type P5*_flhDC_* (TATGCT) to TCTGCC (P5^−^.2*_flhDC_*) or TCTGCT (P5^−^.3*_flhDC_*) but not to TACGCT (P5^−^.4*_flhDC_*) reduced significantly the transcription of *flhDC* to the same extent as the mutation of −10 to GTTGGT (P5^−^.1*_flhDC_*) (Figure 4D). These results indicated that P1*_flhDC_* and P5*_flhDC_* are bona-fide promoters.

Analysis of mutations of −10 sequences of the P2*_flhDC_* and P6*_flhDC_* (overlapping with the CRP binding site which is required for the transcription of *flhDC* from P1*_flhDC_* promoter) and P3*_flhDC_* (overlapping with the LrhA binding site) and P4*_flhDC_* were not conclusive (Supplementary Text S1 & Figure S3).

We further investigated the authenticity of the six putative TSSs of the *flhDC* operon, by engineering strains with combined mutations in the promoter region of *flhDC* leaving only one wild-type −10 sequence from the six described promoters. Thus, P1^+^ designates a strain that has only a functional P1 promoter, etc. We also constructed a control strain with combined mutations in all the six promoters, AP^−^ *_flhDC_* (All Promoters mutated). We established that P1^+^ *_flhDC_* and P5^+^ *_flhDC_* were able to promote *flhDC* operon transcription but to a lesser extent to what is observed in the wild-type strain (Figure 4E). The transcription of *flhDC* was totally abolished in strains harboring P2^+^ *_flhDC_*, P3^+^ *_flhDC_*, P6^+^ *_flhDC_* and APs^−^ *_flhDC_*, while P4^+^ *_flhDC_* mutants showed very low level of *flhDC* transcription (1.8% relative to the wild-type strain) (Figure 4E). These results suggested that in the wild-type background P1*_flhDC_* and P5*_flhDC_* are the main promoters driving *flhDC* operon transcription with a marginal activity from the P4*_flhDC_* promoter. Yanagihara et al., 1999; have demonstrated that P6*_flhDC_* is only active in the absence of CRP, we confirmed that P6^+^ *_flhDC_* (only P6 is functional) is inhibited by CRP, because in a *crp* null mutant there was an increase of transcription of P6^+^ *_flhDC_* compared to wild-type (Figure S3H).

Since only mutations in P1*_flhDC_* and P5*_flhDC_* promoters significantly affected the expression of *flhDC*, we would expect the level of transcription of *flhDC* operon in the absence of both P1 and P5 promoters to be similar to the level of transcription of *flhDC* operon in the absence of all *flhDC* promoters (P1 through P6). To investigate this hypothesis, we measured the luciferase activity in a strain with combined mutations in P1*_flhDC_* and P5*_flhDC_* promoters (P1^−^P5^−^ *_flhDC_*) and compared it to the luciferase activity of a wild-type strain and to a strain with all six promoters mutated (strain AP's). We observed that transcription of *flhDC* operon in strain P1^−^P5^−^ *_flhDC_* was totally abolished to the same levels observed in a strain with all *flhDC* promoters mutated (Figure 4F). These results demonstrated that in a wild-type background P1*_flhDC_* and P5*_flhDC_* are the major promoters driving transcription of *flhDC* operon. We concluded that transcription of the *flhDC* operon in strain P1^−^ *_flhDC_* (harboring mutations of the −10 box of P1*_flhDC_*) is driven from the P5*_flhDC_* promoter and that transcription of the *flhDC* operon in strain P5^−^ *_flhDC_* (harboring mutations of −10 box of P5*_flhDC_*) is driven from P1*_flhDC_*.

### Dynamics of *flhDC* transcription from P1*_flhDC_* and P5*_flhDC_* promoters

**Figure 5:**
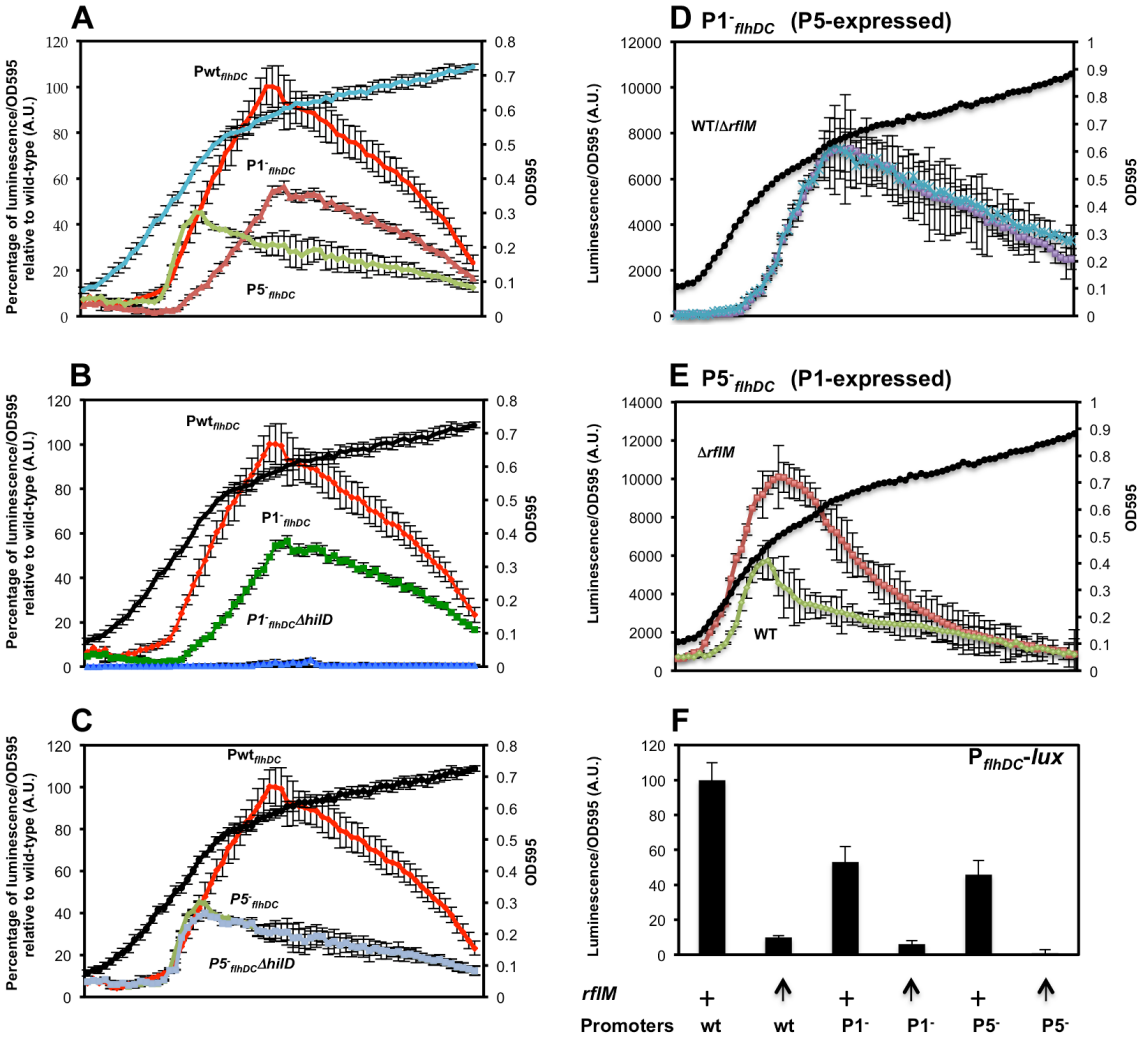
Transcription levels of the P1*_flhDC_* and the P5*_flhDC_* promoters during the cell growth phase and their regulation by HilD and RflM. (**A**) Luciferase activity was measured in three genetic backgrounds: Pwt*_flhDC_-luxCDBAE-*Pwt*_flhDC_flhD^+^C^+^* (TH18684) filled square, P1^−^ *_fhlDC_-luxCDBAE-*Pwt*_flhDC_flhDC^+^* (TH18889) filled circle and P5^−^ *_fhlDC_-luxCDBAE-* Pwt*_flhDC_flhDC^+^* (TH18895) filled triangle. Luciferase activity relative to the wild-type strain is shown (first axis) along with the OD 595 (Second axis). In the absence of the P5 promoter (P5^−^ *_flhDC_*) the *flhDC* operon (transcribed from P1) was activated earlier than the isogenic strain that transcribed *flhDC* from the P5 promoter (P1^−^ *_flhDC_*). Transcription of the *flhDC* operon from the P1 promoter (P5^−^ *_flhDC_*) was activated at the same time as with the wild-type promoter (Pwt*_flhDC_*) at OD∼0.2. When cells reach an OD of 0.4, P1*_flhDC_* promoter activity (P5^−^ *_flhDC_*) ceased and declined afterwards. Transcription from the P5*_flhDC_* start-site (P1^−^ *_flhDC_*) took place at an OD of ∼0.35. (**B & C**) HilD promotes transcription of the *flhDC* operon from the P5*_flhDC_* promoter. Luciferase activity relative to the wild-type strain is shown (First axis) along with the OD 595 (Second axis). Luciferase activity was investigated in five strains: Pwt*_flhDC_-luxCDBAE-*Pwt*_flhDC_flhD^+^C^+^* (TH18684), P1^−^ *_fhlDC_-luxCDBAE-*Pwt*_flhDC_flhD^+^C^+^* (TH18889), P1^−^ *_fhlDC_-luxCDBAE-*Pwt*_flhDC_flhD^+^C^+^* Δ*hilD*::TetRA (TH19965), P5^−^ *_fhlDC_-luxCDBAE-*Pwt*_flhDC_flhD^+^C^+^* (TH18895) and P5^−^ *_fhlDC_-luxCDBAE-*P_wt_ *flhD^+^C* ^+^ Δ*hilD*::*tetRA* (TH19966). Transcription of *flhDC* operon from the P5*_flhDC_* promoter (P1^−^ *_flhDC_*) was totally abolished in the absence of HilD. The absence of HilD had no effect on the transcription of *flhDC* operon from the P1*_flhDC_* promoter (P5^−^ *_flhDC_*). Each data point of the plots represents the average of five independent replicates performed in different days of six measurements for wild-type *flhDC* promoter and three measurements for the rest of strains. (**D & E**) RflM feedback inhibits transcription of the *flhDC* operon. Luciferase activity of P1^−^ *_flhDC_* and P5^−^ *_flhDC_ flhDC* promoters expressing the *luxCDBAE* reporter is presented as a function of the cell growth phase in isogenic strains in the presence (WT) and absence (Δ*rflM*) of RflM. Luciferase levels at different points during the cell growth phase were measured for the (**D**) P1^−^ *_fhlDC_-luxCDBAE-*Pwt*_flhDC_flhD^+^C^+^*, and (**E**) P5^−^ *_fhlDC_-luxCDBAE-*Pwt*_flhDC_flhD^+^C^+^* duplication constructs. Growth conditions and luciferase activity were analyzed as described in Figure 2. A representative growth curve is shown in each plot. Plots represent the average of five independent replicates performed in different days of six measurements for each data point. Error bars represent standard deviation. (**F**) Overproduction of RflM inhibits transcription from the P1*_flhDC_* and P5*_flhDC_* promoters. Luciferase activity for Pwt*_fhlDC_-luxCDBAE-*Pwt*_flhDC_flhD^+^C^+^*, P1^−^ *_fhlDC_-luxCDBAE-*Pwt*_flhDC_flhD^+^C^+^* and P5^−^ *_fhlDC_-luxCDBAE-*Pwt*_flhDC_flhD^+^C^+^* was investigated, when cells reached an OD 1, in two genetic backgrounds: P*_araBAD_FCF*, and P*_araBAD_rflM* ^+^. Growth conditions and luciferase activities were analyzed as described in Figure 4. To induce expression of *rflM* from the arabinose promoter (P*_araBAD_rflM* ^+^), arabinose was added at 0.2% (indicated by an arrow). (+) Indicates wild-type level of *rflM*. Chart represents the average of three independent experiments.

**Figure 6:**
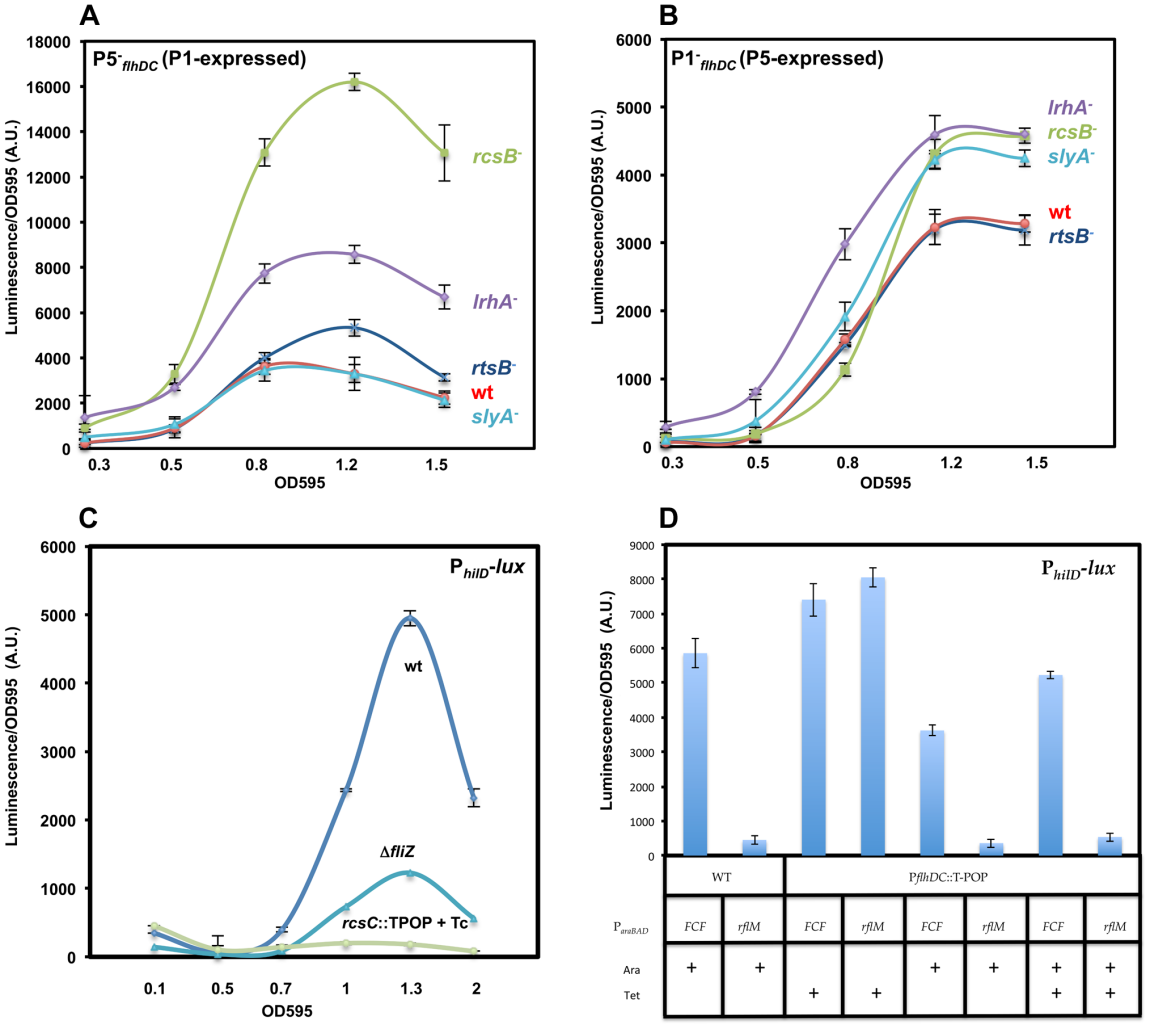
Effects of RcsB, LrhA, RtsB and SlyA on transcription of P1*_flhDC_* and P5*_flhDC_*. For these assays, we compared the transcription from the P1^−^ *_flhDC_* (defective in the P1 start-site) and the P5^−^ *_flhDC_* (defective in the P5 start-site) promoter constructs. Plots represent luciferase activity divided by the OD595 plotted against the OD595 values shown at the bottom of the chart. (**A**) RcsB, LrhA and RtsB but not SlyA repressed transcription from the P1*_flhDC_* promoter. Luciferase activity of P5^−^ *_fhlDC_-luxCDBAE-*Pwt*_flhDC_flhD* ^+^ *C* ^+^ transcriptional fusion (P1-expressed) was investigated in five genetic backgrounds: wild-type (TH18895), Δ*rcsB*::*tetRA* (TH20237), *rtsB*::T-POP (TH19976), Δ*lrhA*::*tetRA* (TH19974), *slyA*::T-POP (TH19975). (**B**) RcsB, LrhA and SlyA but not RtsB are negative regulators of P5*_flhDC_* promoter. Luciferase activity of P1^−^ *_fhlDC_-luxCDBAE-*P_wt_ *flhD* ^+^ *C* ^+^ transcriptional fusion (P5-expressed) was measured in wild-type (TH18889), Δ*rcsB*::*tetRA* (TH20236), *rtsB*::T-POP (TH19972), Δ*lrhA::tetRA* (TH19970) and *slyA*::T-POP (TH19971)., (**C**) RcsB inhibits *hilD* transcription in an *flhDC* independent manner. Luciferase activities of the P*_hilD_*-*luxCDBAE* transcriptional fusion in wild-type (*rcsC* ^+^) (TH19425), *rcsC*::T-POP (TH19687) and Δ*fliZ*::FCF (TH19690) backgrounds were recorded as described in Figure 1. FliZ, a post-translational activator of HilD, promotes transcription of the auto-regulated *hilD* gene. Tetracycline (Tc) was used at 3 µg/ml to induce *rcsC* transcription in the *rcsC*::T-POP background resulting in activation of RcsB. Upon RscB acticvation (*rcsC*::T-POP +Tc), transcription of *hilD* was abolished. The inhibitory effect of RcsB on *hilD* transcrption (40-fold) is more dramatic than the four-fold decrease in the absence of FliZ. Results are the average of two independent experiments performed in duplicate. Error bars represent standard deviation. (**D**) RflM inhibits *hilD* transcription in an *flhDC* independent manner. Luciferase activity of strains harboring a *hilD* transcriptional fusion, P*_hilD_*-*luxCDBAE*, was measured in four genetic backgrounds, P*_araBAD_*::FCF (TH20541) (Column 1), P*_araBAD_::rflM* ^+^ (TH20542) (Column 2) and P*_araBAD_*::FCF P*_flhDC_*::T-POP (TH20543) (Column 3, 5 and 7) and P*_araBAD_::rflM* ^+^ P*_flhDC_*::T-POP (TH20544) (Column 4, 6 and 8). P*_araBAD_::rflM* ^+^ strains, in the presence of arabinose (Ara and +) leads to the overexpression of *rflM and* P*_araBAD_::FCF* serves as a control. Addition of tetracycline (Tet and +) to *P_flhDC_::T-POP* strains allows the overexpression of *flhDC* and in the absence of tetracycline the cells are *flhDC^−^*. Cells were diluted 1 to 500 from an overnight culture into LB in the presence arabinose, tetracycline or arabinose and tetracycline. 0.2% arabinose (Ara) was added to induce transcription of *rflM* and 3 µg/ml tetracycline (Tet) to induce transcription of *flhDC*. At an OD595∼1, the luciferase activity was recorded as described in Figure 4.

Once we established that P1*_flhDC_* and P5*_flhDC_* are the main promoters driving transcription of the *flhDC* operon, we monitored the expression of the P1*_flhDC_* and P5*_flhDC_* promoters at different optical densities using Pwt*_flhDC_*, P1^−^ *_flhDC_* and P5^−^ *_flhDC_* constructs (Figure 5A). The transcription profile of *flhDC* operon in strains P1^−^ *_flhDC_* (P5-expressed) and P5^−^ *_flhDC_* (P1-expressed) demonstrated that both promoters are required for transcription of *flhDC* because the expression of *flhDC* operon in constructs P1^−^ *_flhDC_* (P5-expressed) and P5^−^ *_flhDC_* (P1-expressed) did not reach the expression levels of the wild-type strain, Pwt*_flhDC_* (both P1 and P5 are expressed) (Figure 5A). Moreover, transcription of *flhDC* operon from P1*_flhDC_* is activated earlier than P5*_flhDC_* because (i) the transcription profile of the *flhDC* operon in construct P5^−^ *_flhDC_* (P1-expressed) overlapped with that of the wild-type strain from OD 0.1 to OD 0.4, (Figure 5A) and (ii) there was a delay in the transcription of *flhDC* operon in construct P1^−^ *_flhDC_* (P5-expressed) where the transcription started taking place at OD∼0.35 (Figure 5A) compared to the wild-type Pwt*_flhDC_* and P5^−^ *_flhDC_* (P1-expressed) strains (OD∼0.2). The same hierarchy of expression of P1 and P5 was observed in batch culture (Figure 6A & B). The transcription of *flhDC* operon in P5^−^ *_flhDC_* (P1-expressed) started declining at OD∼0.4–0.5, meanwhile, transcription of *flhDC* operon in strain P1^−^ *_flhDC_* (P5-expressed) was more pronounced at a later growth stage accounting for ∼60% relative to the wild-type at OD∼0.6 (Figure 5A). It is apparent from the dynamic profile of *flhDC* operon transcription, that P5*_flhDC_* promoter transcription occurs concomitantly with a cessation or decline in the transcription from P1*_flhDC_* (Figure 5A). These results indicate that P1*_flhDC_* is an early promoter, whose activation drives the transcription of *flhDC* operon synthesis at early growth phase followed by a cessation or decline once P5*_flhDC_* promoter is activated.

### HilD specifically activates transcription from the P5*_flhDC_* promoter

We have demonstrated that HilD is a positive regulator of *flhDC* transcription (Figure 1D & 2D). As shown (Figure 2D), when cells are grown in the 96 well plate format, the effect of HilD on the transcription of *flhDC* takes place starting at OD∼0.4. In order to determine which of the two promoters, P1*_flhDC_* or P5*_flhDC_*, is the target of the positive regulation by HilD, we compared the dynamic profile of *flhDC* transcription in Pwt*_flhDC_*, P1^−^ *_flhDC_* and P5^−^ *_flhDC_* constructs in a wild-type and its isogenic strain *hilD* null mutant (Figures 5B & C). We established that, relative to the wild-type strain background, a deletion of *hilD* (i) reduced Pwt*_flhDC_* promoter transcription; (ii) abolished the transcription of *flhDC* operon in construct P1^−^ *_flhDC_* (P5-expressed) (Figure 5B) and (iii) did not affect the transcription of *flhDC* operon in construct P5^−^ *_flhDC_* (P1-expressed) (Figure 5C). These results indicate that HilD promotes transcription from P5*_flhDC_* and has no apparent effect on P1*_flhDC_* promoter transcription.

### The negative autoregulation of *flhDC* transcription via RflM is at the P1*_flhDC_* promoter

Transcription of the *flhDC* operon is subjected to negative feedback by RflM, which is activated at the transcriptional level by FlhD_4_C_2_ [21]. To further study the effect of the negative autoregulation on *flhDC* operon transcription kinetics, we monitored the transcription profile, over time, in the three strains Pwt*_flhDC_*, P1^−^ *_flhDC_* and P5^−^ *_flhDC_* in the absence and presence of RflM. We established that there was an increase in the transcription from Pwt*_flhDC_* in the absence of RflM (Figure 2B). We demonstrated that the P1*_flhDC_* promoter is under negative autoregulation by RflM because the expression of *flhDC* operon in strain P1^−^ *_flhDC_* (P5-expressed) was similar between the wild-type and its isogenic *rflM* null mutant (Figure 5D). Additionally, we found that RflM did not appear to regulate P5*_flhDC_* because *flhDC* transcription in strain P5^−^ *_flhDC_* (P1-expressed) increased in the absence of RflM (Figure 5E). These results demonstrated that in the wild-type background the P1*_flhDC_* promoter is subjected to negative autoregulation through RflM, while transcription from P5*_flhDC_* appeared to be RflM independent.

We employed an alternative approach to confirm which of the *flhDC* promoters is specifically inhibited by the transcriptional factor RflM. We monitored the transcription of *flhDC* in a strain that overproduces RflM under control of the arabinose promoter, P*_araBAD_*::*rflM* ^+^. In the presence of arabinose, used to induce overexpression of *rflM*, we observed an inhibition of transcription of *flhDC* operon in the three strains tested, Pwt*_flhDC_*, P1^−^ *_flhDC_* and P5^−^ *_flhDC_* (Figure 5F). These results suggest that RflM is able to inhibit transcription of *flhDC* operon from both promoters, P1 and P5, which is in contradiction to the specific inhibition of the P1*_flhDC_* but not the P5*_flhDC_* promoter by RflM observed in Figures 5D & E. RflM protein production or stability appears to decline in function of cell growth cycle (Figure 3A), suggested that continuous production of RflM might affect indirectly the expression of P5*_flhDC_*. Because HilD is an activator of the P5*_flhDC_* promoter, we hypothesized that overexpression of Rfl*M* inhibits transcription of *hilD* gene. In order to test this hypothesis, we monitored the activity of a luciferase transcriptional fusion of the *hilD* promoter, P*_hilD_*, in two genetic backgrounds: (i) P*_araBAD_*::*FCF* (ii) P*_araBAD_*::*rflM* ^+^. We observed that under conditions that overproduce RflM, presence of arabinose, there was an inhibition of transcription of the autoregulated gene *hilD* (Figure 6, compare column 1 to column 2). Note that the strains used to determine luciferase activity are all *flhD* ^+^C^+^, and overexpression of RflM inhibits *flhDC* transcription required for production of the posttranslational regulator of HilD. Thus, the effect of RflM, on *hilD* transcription could be indirect through inhibiting *flhDC*. To test if the effect of RflM on *hilD*, is direct or indirect we used two additional strains (i) P*_araBAD_*::*FCF* P*_flhDC_*::T-POP and (ii) P*_araBAD_*::*rflM* ^+^ P*_flhDC_*::T-POP. For the P*_flhDC_*::T-POP backgrounds the *flhDC* operon is transcribed from the tetracycline(Tc)-inducible *tetA* promoter, and as such are *flhDC* ^−^ in the absence of tetracycline and *flhDC* ^+^ in the presence of tetracycline. First, we observed that *flhDC* controlled transcription of the *hilD* gene, because in the absence of Tc, there was a 2-fold decrease in the P*_hilD_* transcription level in the P*_flhDC_*::TPOP strain background (Figure 6D, compare column 3 to column 5). Moreover, we demonstrated that under condition of RflM overexpression, there was a higher level of inhibition of *hilD* transcription compared to the reduction observed in the P*_flhDC_*::T-POP background (Figure 6D, compare column 5 to column 6). The overproduction effect of RflM was not rescued by addition of Tc to induce *flhDC* transcription, an activator of *hilD* transcription (Figure 6D, compare column 6 to column 8). These results demonstrated that RflM could inhibit transcription of the *hilD* gene in an *flhDC* independent manner. Thus *flhDC* and *rflM* have opposite effects on the transcription of *hilD*, where *flhDC* is an indirect positive regulator of HilD, yet high levels of RflM inhibit *hilD* transcription. Since HilD is an activator of P5*_flhDC_* transcription, we conclude that the negative effect of RflM overproduction on transcription of P5*_flhDC_* is indirect and through inhibition of *hilD* gene transcription,

### Targeting of the *flhDC* promoter region by RcsB, LrhA, SlyA and RtsB

The presence of two principal TSSs within the *flhDC* operon promoter region combined with the hierarchical regulation by different transcriptional factors, suggests that there is differential regulation at the promoter by different transcriptional regulators at different cell growth phases. We investigated which of the specific regulators: RcsB, LrhA, SlyA and RtsB control transcription of *flhDC* through the P1*_flhDC_* and P5*_flhDC_* promoters start-sites.

#### 1. P1*_flhDC_* is negatively regulated by RcsB, RtsB and LrhA but not by SlyA

To determine if RcsB, RtsB, LrhA and SlyA regulate P1*_flhDC_*, we monitored the transcription of *flhDC* operon of the construct P5^−^ *_flhDC_* (P1-expressed) in strains defective in either the *rcsB*, *lrhA*, *slyA* or *rtsB* genes. We observed increased transcription in the P5^−^ *_flhDC_* background, in either *rcsB*, *rtsB* or *lrhA* null mutants compared to the wild-type strain (Figure 6A). The transcription of P1*_flhDC_* increased 5-fold, 2-fold and 1.6-fold in *rcsB*, *lrhA* and *rtsB* mutant strains, respectively. These results demonstrated that RcsB, LrhA and RtsB repress transcription from P1*_flhDC_*. However, a null mutation in *slyA* gene did not affect transcription from P1*_flhDC_*, because there were no differences in the transcription levels for the P5^−^ *_flhDC_* mutant promoter at any point of time during all the growth phases between the wild-type and the *slyA* mutant. The same effect of RcsB, LrhA and SlyA was also observed in strain P1^+^ *_flhDC_* (this strain is (P5P4P3P6P2)^−^) (Figure S4.A). However, there was no effect of *rtsB* mutation on the expression of P1^+^ *_flhDC_*, which could be attributed to either the low level of expression *flhDC* in construct P1^+^ *_flhDC_* or to the additional mutations (P5P4P3P6P2)^−^ present in the P1^+^ *_flhDC_* construct (See supplementary Text S1).

#### 2. P5*_flhDC_* is negatively regulated by RcsB, LrhA, SlyA but not by RtsB

We monitored the transcription of the *flhDC* operon in construct P1^−^ *_flhDC_* (P5-expressed) in strains lacking either the *rcsB*, *lrhA*, *slyA* or *rtsB* genes. We demonstrate that the transcription from P5*_flhDC_* promoter is regulated by RcsB, LrhA and SlyA proteins, because transcription of *flhDC* in construct P1^−^ *_flhDC_* (P5-expressed) increased in *rcsB* (2-fold), *lrhA (2-*fold) and *slyA* (1.8-fold) mutant strains (Figure 6B). We also demonstrated that transcription of the P5*_flhDC_* promoter is not regulated by RtsB protein because transcription of *flhDC* in construct P1-flhDC (P5-expressed) was independent of RtsB (Figure 6B). In strain P5^+^ *_flhDC_*, (this strain is (P4P3P6P2P1)^−^), we observed the same regulation as with P1^−^ *_flhDC_* (Figure S4.B).

The transcription kinetics of the P1^−^ *_flhDC_* (P5-expressed) in the Δ*rcsB* mutant was different than that of the Pwt*_flhDC_* or the P5^−^ *_flhDC_* (P1-expressed) in the absence of RcsB. While, there was a relief in the inhibition of transcription for the Pwt*_flhDC_* or P5^−^ *_flhDC_* (P1-expressed) constructs at earlier time points of cell growth, the *rcsB* mutation resulted in increased transcription in the construct P1^−^ *_flhDC_* (P5-expressed) only at later stage of growth (Figure 6B). It has been demonstrated that RcsB regulates *flhDC* transcription by direct binding to an RcsB binding sequence located 11 bp downstream of P1*_flhDC_*. Inspection of the DNA upstream region of *flhDC* operon did not reveal the presence of any additional consensus RcsB-binding site, suggesting that the inhibitory effect of RcsB on transcription from the P5*_flhDC_* promoter might be indirect and through the repression of an activator or activation of a repressor. It has been shown that RcsB inhibits *hilA* transcription [61], whose activation is under the control of *hilD*. Because *hilD* is an activator of transcription from the P5*_flhDC_* promoter, we hypothesized that the effect of RcsB on P5*_flhDC_* transcription was due to derepression of *hilD* transcription in an *rcsB* mutant background. To test this hypothesis, we monitored the expression of a transcriptional fusion of *hilD* to luciferase, P*_hilD_*-*lux*, in wild-type, Δ*fliZ*::FCF and *rcsC*::T-POP strains (Figure 6C). We used an *rcsC*::T-POP allele that results in tetracycline(Tc)-dependent transcription of the *rcsC* gene [62] and thus activation of the transcription factor RcsB, to monitor the effect of RcsB on *hilD* transcription. We used a *fliZ* null mutant to detect if the effect of RcsB on *hilD* is through the *flhDC* regulated gene *fliZ*, which is the post-translational activator of the autoregulated *hilD* gene. We observed that FliZ regulated transcription of *hilD*, because there was a 2-fold reduction of P*_hilD_*-dependent transcription in the strain lacking *fliZ* compared to the *fliZ* ^+^ background (Figure 6C). However, under conditions that over-express RcsC (addition of Tc in the presence of the *rcsC*::T-POP allele to induce transcription of *rcsC*), the transcription of *hilD* was abolished (Figure 6C). Compared to the effect of deleting *fliZ*, overexpression of RcsC exerted a more pronounced inhibitory effect on *hilD* transcription. These results demonstrate that RcsB inhibits transcription of *hilD* in both *flhDC* dependent and independent manners and suggested that the P5*_flhDC_* promoter is indirectly regulated by the RcsB transcriptional factor.

### The timing of transcription of *flhDC* as a prerequisite for motility

**Figure 7:**
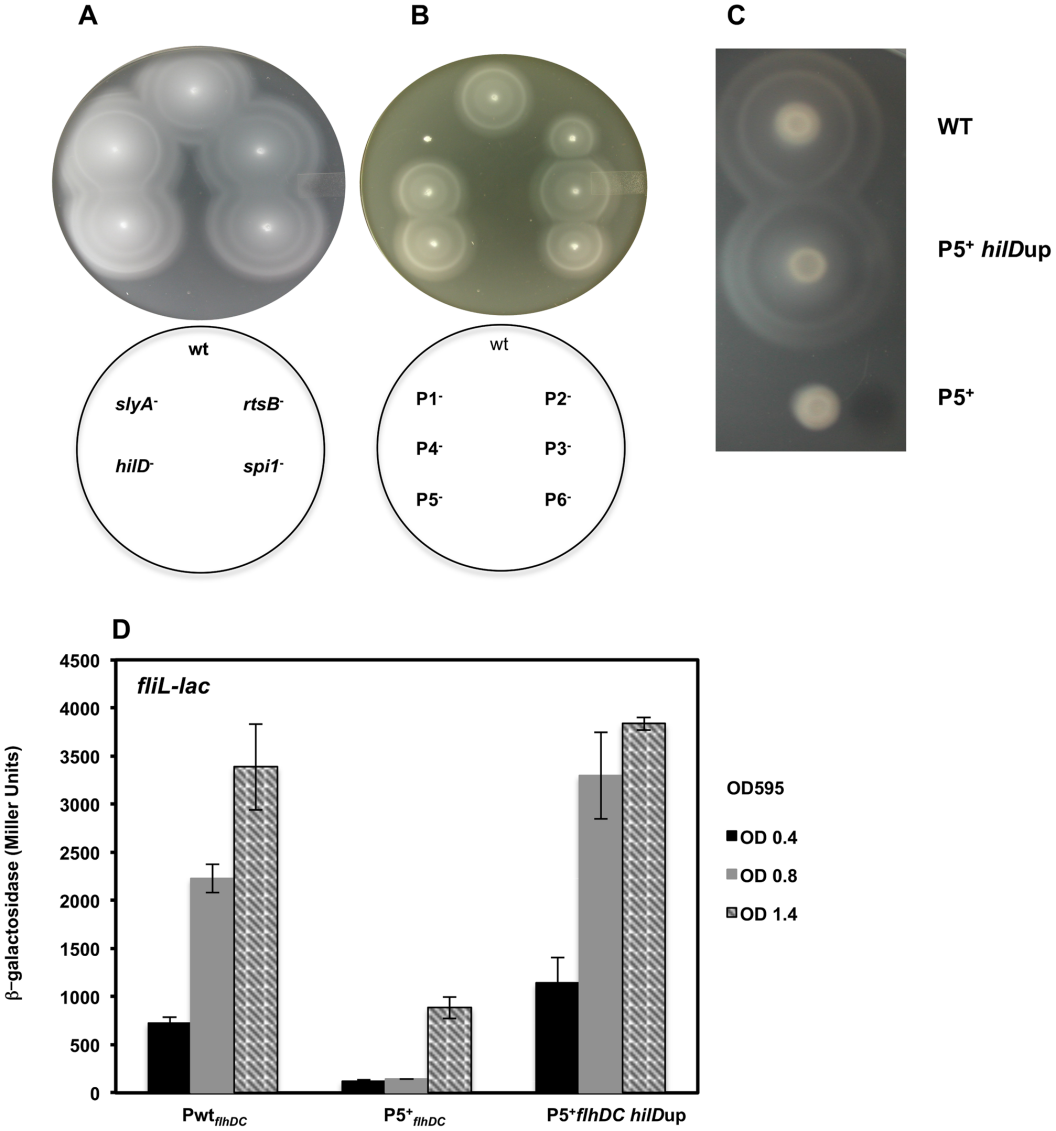
Time-dependent transcription of *flhDC* operon controls motility of *Salmonella*. (**A**) A representative image of motility of the wild-type strain compared to (**A**) *slyA*, *rtsB*, *spi*1 and *hilD* null mutants. Null mutations in *slyA*, *rtsB*, *spi1* or *hilD* does not affect motility compared to the wild-type strain (**B**) A representative image of motility of the wild-type strain compared to constructs harboring single promoters mutations in the *flhDC* regulatory region. (**C &D**) Early transcription of P5*_flhDC_* promotes motility (**C**) the motility defect of P5^+^ construct (only P5 is active and the promoters P1, P2, P3, P4 and P6 are mutated) was rescued by a mutation that overexpresses *hilD* (P5^+^ *hilD*up) and (**D**) transcription of class 2 gene, *fliL*, of the P5^+^ construct in a wild-type strain compared to its isogenic strain *hilD*up (mutation that overexpress HilD). ß-galactosidase activity (Miller Units) of a lac fusion to *fliL* gene was investigated in three genetic backgrounds: wild-type, P5^+^ *_flhDC_* and P5^+^ *_flhDC_ hilD*up strains. Values are average of two experiments done in duplicate at different ODs.

There appears to be five stages of *flhDC* transcription that are controlled by three clusters of response regulators. Deletion of either, *rcsB*, *lrhA* or *rflM* resulted in increased motility compared to the wild-type strain [18], [19], [62]. We observed that null mutations in any of the late regulators: *hilD*, *rtsB* or *slyA*, did not affect motility (Figure 7A). Based on the expression profiles of the *flhDC* operon in these mutant strains, these results establish that *Salmonella* wild-type motility will only need to reach a threshold of *flhDC* expression for motility, while increased *flhDC* expression later in the growth phase has no further effect on motility. It is noteworthy to mention that factors that affected the early transcription of the P1*_flhDC_* promoter: LrhA, RcsB (Figure 6A) and RflM (Figure 5E) affected motility while transcriptional factors, HilD and SlyA, that regulate P5*_flhDC_* promoter late in the growth phase (Figure 5B & 6B) did not affect motility (Figure 7A). Moreover, RtsB, by inhibiting transcription from P1*_flhDC_* at later stages of growth (Figure 6B), did not inhibit motility suggesting that the growth phase combined with activation of *flhDC* promoters is important for motility (Figure 7A). It is noteworthy to mention that the factors that affected transcription of P5 *flhDC* but not motility are bona fide virulence factors.

We decided to study the effect of the *flhDC* promoter mutations on the motility of *Salmonella*. We constructed strains harboring single mutation in each of the promoters separately. Thus P1^−^ refers to a strain that has a mutation in P1 promoter, etc. Note that these strains in contrast to strains harboring the luciferase constructs do not harbor a duplication of the *flhDC* operon. We demonstrated that strains defective in P1*_flhDC_* start-site transcription (only P1 is mutated) were non-motile while P5*_flhDC_* defective strains (only P5 is mutated) exhibited no apparent reduction of motility (Figure 7B). There was a motility defect of the strains P2^−^ and P6^−^ that is related to the effect of CRP (as discussed earlier and in Supplementary material). The motility of P3^−^ and P4^−^ were not significantly different from the wild-type strain. These results confirmed that in the wild-type background transcription from P1*_flhDC_* is a prerequisite for motility while P5*_flhDC_* is not required for motility. These results also suggested that the right timing of expression of *flhDC* is essential for motility. If this hypothesis is correct, we could expect that if *flhDC* is expressed from P5*_flhDC_* promoter at an early time point it should confer a motility phenotype. To test this hypothesis we used the non-motile strain P5^+^ (only P5 is functional and the other promoters are mutated) (Figure 7C) to isolate suppressors of motility inhibition. This strain was used in order to limit isolating mutations in the other promoters of *flhDC* that would otherwise suppress motility [16]. We isolated a spontaneous suppressor that restores motility to the P5^+^ strain (Figure 7C) and mapped the mutation to the promoter region of *hilD* gene (addition of a thymine residue at position −51 from the start codon of HilD and resulting in higher expression of *hilD* (labeled *hilD*up)). The isolation of this mutation confirmed that HilD regulates the P5*_flhDC_* promoter. If the hypothesis that the timing of expression of *flhDC* as a prerequisite for motility is correct, then a *hilD*-up mutation should promote transcription of *flhDC* operon from P5 promoter at early growth phase. To test this hypothesis we used a transcriptional *lac* fusion to *fliL*, a class 2 promoter that is positively regulated by FlhD_4_C_2_, as readout to determine the expression of the P5 promoter transcription. The transcription of *fliL* indicates the presence of FlhD_4_C_2_-dependent transcription. Transcription of *fliL* in the P5^+^ strain was very low during early growth phases and increased when cells reached an OD of 1.4 (Figure 7D). These results suggest that P5^+^ cells are able to express flagellar genes at later stage of cell's growth phase yet they are not motile. Interestingly, overexpression of *hilD*, *hilDup* mutant resulted in a premature activation of P5*_flhDC_*, leading to the transcription of *fliL* at early growth phase and similar to the timing and levels of the wild-type strain (Figure 7D). These results suggested that the timing of FlhD_4_C_2_ production during an early growth phase is critical for motility.

## Discussion

The complex networks and the number of factors necessary for the production of functional flagella and the resulting motility, though beneficial for the bacteria, represent a significant requirement on the cell's resources [63], [64]. At the top of this cascade sits the *flhDC* operon [7]. We established now that *Salmonella flhDC* operon is primarily transcribed from two promoters, P1*_flhDC_* and P5*_flhDC_*. The activities of these two promoters are coupled to five stages controlling *flhDC* transcription and each stage is differentially controlled by a set of transcriptional factors: (1) repression of transcription of *flhDC* during the initial growth phase by LrhA and RcsB (2) repression by RflM at early exponential phase (3) activation through the action of HilD at mid exponential phase (4) repression by SlyA and RtsB at the onset of stationary phase, and finally (5) shut down at late stationary phase.

### Dynamics of *flhDC* operon transcription in a growth phase dependent manner

The pre-log steady state transcription of *flhDC* regulation is controlled by two transcription factors, RcsB and LrhA. Null mutation in any of these transcriptional regulators, promoted *flhDC* transcription early in the growth phase and this inhibition was maintained throughout the rest of the growth phase (exponential and stationary). We found that the effect of LrhA and RcsB was coincident with activation of transcription of their respective genes. As cell densities reached an OD of 0.2–0.3, transcription of *flhDC* increased. The increased *flhDC* transcription resulted in transcription of *rflM*, which in turn resulted in the feedback inhibition of *flhDC* transcription. This effect was consistent with the concurrent transcriptional activation of *flhDC* and *rflM*, where a surge of transcription of *rflM* mimicked that of *flhDC* and decayed quickly compared to the rest of the regulators controlling *flhDC* transcription. At the protein level, RflM appeared to follow the same early production and a quick decay as observed at the transcriptional level. We conclude that RflM limits *flhDC* transcription perhaps to efficiently control the kinetic expression of the middle and late flagellar class genes to facilitate flagellum assembly. Class 2 promoters respond differently to FlhD_4_C_2_ levels allowing the cell to control the timing of an individual class 2 operon transcription with respect to the other class 2 operons. Auto-repression at the transcriptional level has been shown to reduce relative variance and duration of fluctuations, and consequently limits noise in downstream expression [65], [66]. Expression of *fliC*, encoding the filament component of the flagellum, has been demonstrated to be bistable [67], [68]. We suggest that RflM would fulfill the noise reduction of flagellar class 2 and class 3 promoters transcription during exponential growth phase, by controlling class 1 *flhDC* operon transcription. In support of this hypothesis, a null mutation of *rflM* gene has been shown to increase heterogeneity of *fliC* expression in a cell population when compared to wild-type [21].

Once bacteria reach mid-exponential phase growth, there is a second layer of control on *flhDC* operon transcription. This control is positive, and is brought on by the effect of a virulence-associated transcription factor, HilD. There was a delay in the positive effect of HilD compared to the negative control exerted by RcsB, LrhA and RflM. This delayed HilD effect on *flhDC* operon transcription was due to the time required to activate HilD expression through FlhD_4_C_2_-dependent FliZ production. FlhD_4_C_2_ activates *fliZ* gene transcription from a flagellar class 2 promoter and FliZ, in turn, activates *hilD* expression at the post-translational level [57]. Finally, a third layer of *flhDC* transcription takes place and, unexpectedly, is also controlled by HilD. HilD activates the transcription of two regulatory factor genes, *rtsB* [17] and *slyA* (data not shown). RtsB and SlyA are two DNA binding regulators, which then act to inhibit *flhDC* transcription.

There is no doubt that flagellar motility provides a significant survival advantage over non-motile bacteria in many environmental situations. Furthermore, the link between production of flagella and other regulatory networks [69]–[72] would be affected if an unchecked production of flagella occurs. The overexpression of the flagellar regulon also attenuates *Salmonella* virulence [73]. These observations could explain the array of negative regulators controlling transcription of *flhDC* operon and keeping a check on the flagellar synthesis as well as FlhD_4_C_2_ production.

### P1*_flhD_* and P5*_flhD_* are the main promoters driving *flhDC* transcription

While the literature reports the presence of either four or six transcription start-sites in the *flhDC* promoter region [13], [29], our work suggests that only the P1*_flhDC_* and P5*_flhDC_* promoters are functional in a wild-type strain under laboratory growth conditions. First, we demonstrated that there was a reduction in *flhDC* operon transcription in the absence of P1*_flhDC_* or P5*_flhDC_* compared to the wild-type strain (Figure 4C & D). Second, we showed that *flhDC* operon transcription was totally abolished in P1^−^P5^−^ *_flhDC_* double mutant (Figure 4F). We confirmed that the P6*_flhDC_* promoter is active only in the absence of CRP [13]. Moreover, there was no apparent effect of P4*_flhDC_*, P3*_flhDC_* and P2*_flhDC_* promoters on *flhDC* transcription. In *E. coli*, CsrA, a carbon storage global regulator, activates *flhDC* expression in an RNaseE-dependent manner through protection of 5′end cleavage [23]. The 5′-UTR of the P5*_flhDC_* start-site transcript is 534 bases in length. We suspect that the presumed P3*_flhDC_* and P2*_flhDC_* start-sites resulted from RNAseE-dependent RNA-processing and/or degradation of the P5*_flhDC_* transcript. The P4*_flhDC_* start-site might also result from RNA processing; however, the isolation of mutants in the −10 region of P4*_flhDC_* that result in increased *flhDC* transcription suggests there might be unknown conditions where transcription from P4*_flhDC_* occurs [16].

### Activation of *flhDC* operon transcription from the P1*_flhDC_* promoter establish two disparate regulatory loops

Genes with multiple transcription start-sites combined with auto-regulatory networks have been described in other systems. These include, *Salmonella phoP*, *Bordetela pertussis bvgA, E.coli rrnA, and Salmonella fliAZ operon* [27], [74]–[78]. These four cases bear similarity with *flhDC* operon transcription from P1*_flhDC_* and P5*_flhDC_* promoters. However, the case of *flhDC* is more elaborate, where two disparate pathways are used as feedback control. First, we demonstrated a sequential activation of P1*_flhDC_* and P5*_flhD_* transcripts that are growth phase dependent (Figure 5A). The P1*_flhDC_* promoter activating two regulatory pathways resulting in both a negative and a positive regulatory loop and each of these loops has a specific effect on the *flhDC* operon promoters. The negative loop starts with P1*_flhDC_*, leading to the production of FlhD_4_C_2_ that activates *rflM*, which in turn feedback inhibits the P1*_flhDC_* promoter (Figure 5E). The positive feedback loop is also generated from P1*_flhDC_*, where transcription of *flhDC* operon from P1*_flhDC_* leads to *fliZ* gene transcription followed by FliZ activation of *hilD*. HilD then activates the second *flhDC* transcriptional cycle from P5*_flhDC_* (Figure 5B). Paradoxically, HilD controls transcription of *rtsB* and *slyA* genes, whose products binds to the *flhDC* promoter region (Figure 3B) and inhibit transcription, from P1*_flhDC_* and P5*_flhDC_*, respectively (Figures 6A & B).

### Importance of timing of *flhDC* transcription activation on motility and virulence

The three promoter classes of the flagellar regulon, class 1, class 2 and class 3; are expressed in a temporal cascade that coincides with flagellum assembly [79]. The control of flagella production is ultimately determined through the production of FlhD_4_C_2_. However, when *flhDC* is highly over-expressed the cells are not motile for reasons that are not understood. Thus, an intricate temporal control of gene expression and specific quantities of a functional FlhD_4_C_2_ master regulator are essential for motility. For example, the activator of type I fimbriae gene expression, FimZ, represses *flhDC* transcription suggesting that adherence is impeded in the presence of functional flagella. Neither deletion of *flhDC* nor over-expression of *flhDC* affect type I fimbriae gene expression suggesting that the presence of fimbriae (at wild-type levels) does not impede swimming. FlhD_4_C_2_ activity is also required in other cell processes such as Spi1 gene expression and other genes less characterized such as the *srfABC* operon [80], which is implicated in surfactin production and the *modABC* operon [80], which is involved an anaerobic respiration. This leads us to speculate that P1*_flhDC_* is required for flagella production and P5*_flhDC_* is required for growth in various environmental conditions such as in biofilms or in host cells. One possibility is that the activation of *flhDC* transcription from P5*_flhDC_* might represent a mechanism of protein amplification by a surge of transcription, when it is necessary to turn on the Spi1 regulatory network, even under conditions where flagella synthesis is inhibited at the level of *fliA* and *fliC*. This scenario can be very useful after infection when the bacteria requires expression of virulence factors to survive the physical and immune clearance of the eukaryotic host.

Flagella appear to be required for reaching and selecting point of entry of bacteria into host cells [81]. The low pH of the stomach will cause flagella already present to depolymerize [82]. In the intestine, the early transcription of *flhDC* operon from the P1 promoter provides the transcription factor, FlhD_4_C_2_ for expression of functional flagellar machinery to reassemble filaments and allow bacterial cells to swim to selected points of entry into epithelia cells. At the time of invasion, expression of both T3SS1 and flagella has been shown to be required. Thus, in the second step, the already expressed *flhDC* from P1*_flhDC_* promoter activates transcription of *fliZ*, the posttranslational regulator of HilD. In turn, HilD promotes transcription of Spi1 genes, leading to invasion. Thus P1-expressed *flhDC* fulfills two functions: driving the cells near the point of entry and also boosting the expression of Spi1, necessary for invasion, through its effect on HilD. It is noteworthy to mention that invasion of epithelial cells is a rapid process occurring within 10 to 15 minutes after introduction of *S. typhimurium* into the intestinal lumen [83]. Translocation of bacteria across the epithelial barrier and into the underlying tissue is observed within 2 hours after infection of ligated ileal loops [83], [84]. Interestingly *Salmonella* can replicate within two distinct intracellular environments: intravacuolar and cytosolic [85]. Once inside the host, the expression of both flagella and Spi1 appear to be downregulated but not abolished with most of the cytosolic population expressing both flagella and Spi1 at latter stage of infection. In addition, only a subset of T3SS1-induced cytosolic bacteria was motile [85]. We speculate that once bacteria invade epithelial cells, HilD activates P5*_flhDC_* and down-regulates the transcription of P1*_flhDC_* in an RtsB-dependent manner. The transcription from P5*_flhDC_* is bistable leading to two populations of cells, one is flagellated and the other is not (∼10% of cells being flagellated). This bistable expression of P5*_flhDC_* is reminiscent with the bistable expression of Spi1. We suggest that the presence of two populations inside epithelial cells could be explained by the bistability from P5*_flhDC_* promoter and the consequent downregulation of P1*_flhDC_* might represent a mechanism to limit the number of flagellated cells. The cytosolic growth of *Salmonella* leads to the extrusion of epithelial cells as a host defense mechanism [85]. The consequent release of the invasion-prone flagellated cells bacteria back into the mucus rich and inflamed gut endows *Salmonella* with a fitness advantage to use the energy-taxis mechanism to benefit from inflammation [86]. We speculate that the different timing of expression of flagellar promoters P1 and P5 and the bistable expression of P5*_flhDC_* represent a mechanism by which bacteria can disseminate and increase transmission by fecal shedding. These hypotheses are under investigation.

An additional scenario is that the transcription from P5*_flhDC_* has no effect on the synthesis of flagella but rather leads to the production of single subunits of the active transcriptional complex FlhD_4_C_2_. It has been shown that the inhibition of FlhD_4_C_2_-dependent transcription inside host cells is due to the effect of YdiV-mediated ClpXP degradation of the FlhD_4_C_2_ complex. The expression from P5*_flhDC_* late during cell growth will not allow for motility because the activation of the ClpXP leads to the degradation of the complex. However, ClpXP in addition to degrading the FlhD_4_C_2_complex also degrades the FlhC single subunit but not FlhD. This leads to the hypothesis that single FlhD or FlhC subunits might activate transcription of other genes required for virulence [87]

### Conclusions

**Figure 8:**
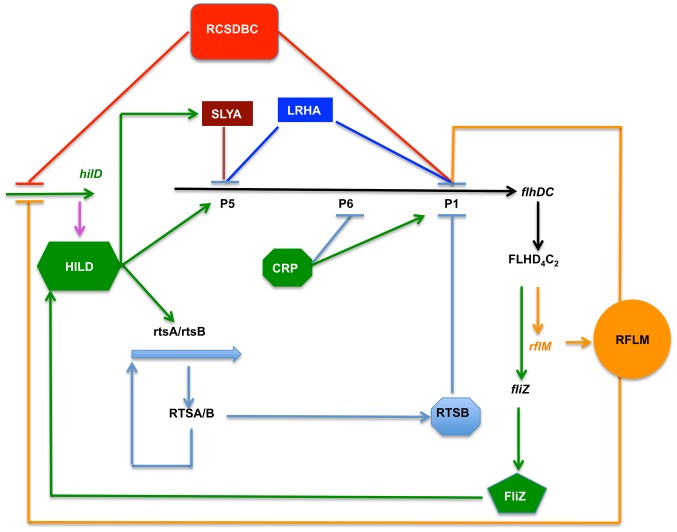
Model depicting the flagellar and Spi1 regulatory circuitry. RcsB and LrhA inhibit transcription of *flhDC* at early cell's growth phase. These two regulatory factors inhibit transcription from the P1*_flhDC_* and P5*_flhDC_* promoters. Under proper conditions CRP activates transcription from P1*_flhDC_*. This activation produces enough FlhD_4_C_2_ to promote synthesis of flagellar proteins required for flagellum assembly and motility of *Salmonella*. There is a simultaneous activation of the FlhD_4_C_2_-dependent *rflM* gene. RflM feedback inhibits any further surge of transcription from P1*_flhDC_*. This effect limits the *flhDC* expression resulting in differential expression of flagellar class 2 genes. RflM transcription appears to be short-lived as there is a quick decay of *rflM* transcription and RflM production. The mechanism by which transcription inhibition of *rflM* happens is unclear. It appears that if RflM expression is maintained, P5*_flhDC_* transcription is not activated. On the other hand, FlhD_4_C_2_ promotes *fliZ* transcription, whose product activates HilD at the posttranslational level. HilD positively activates transcription from P5*_flhDC_* and inhibits P1*_flhDC_* transcription through the activation of *rtsB*. HilD also activates transcription of *slyA*, whose product inhibits transcription from P5*_flhDC_*. In the wild-type strain, *flhDC* transcription from P5*_flhDC_* does not affect motility, where only a threshold of *flhDC* transcription is required to promote motility. The timing at which transcription of *flhDC* takes place appears to be a signal for FlhDC regulation of motility. However, P5*_flhDC_* also is able to promote motility in the absence of P1*_flhDC_* and when appropriate conditions are met such overexpression of HilD that allows for an early transcription of *flhDC* operon from P5*_flhDC_*.

Our finding can be rationalized in terms of a model (Figure 8). Two regulatory factors, LrhA and RcsB regulate *flhDC* by inhibiting transcription from P1*_flhDC_* and P5*_flhDC_*. The effect of RcsB is more dominant on P1*_flhDC_* then on P5*_flhDC_*, whereas LrhA represses more strongly P5*_flhDC_* than P1*_flhDC_*. Transcription activation of P1*_flhDC_* by CRP leads to a rapid transcription of *rflM*, which in turn reduces transcription of P1*_flhDC_*, and limits a rapid class 2 and class 3 genes expression. The FlhD_4_C_2_ complex, already produced, allows motility to proceed and also promotes activation of HilD at the posttranslational level through FliZ, ultimately leading to activation of transcription from the P5*_flhDC_* promoter. This positive autoregulation also generates a subsequent inhibition of *flhDC* operon transcription, of both P1*_flhDC_* and P5*_flhDC_* promoters, by two HilD-induced regulatory factors SlyA and RtsB, themselves regulated by different environmental cues. The activation of transcription from P5*_flhDC_* would lead to higher expression of FlhD_4_C_2_. Though not necessary for motility, it could affect expression of HilD. Because, HilD is required for *Salmonella* survival inside host cells, this positive circle of activation might be well suited for virulence.

## Materials and Methods

### Bacterial strains, primers and standard genetic manipulations

Bacterial strains and primers used in this study are listed in Table S1 and Table S2, respectively (Supplementary Information). Bacterial cells were routinely grown in Luria-Bertani (LB) broth and, when necessary, supplemented with appropriate antibiotics at the following concentrations: Kanamycin (5–10 µg/ml), tetracycline (15 µg/ml) in agar plates and for induction of T-POP 3.5 µg/ml). L-arabinose was used at 0.2% (w/v) when needed. Motility agar plates were prepared as described earlier [62]. The generalized transducing phage of *S. typhimurium* P22*HT105/1 int-201* was used in all transductional crosses [88]

### Construction of transcriptional fusions to a luciferase reporter

For the construction of strain TH18684 DUP[(Pwt*_flhDC_8093*-*luxCDBAE*)*Km*(Pwt*_flhDC_-flhD^+^*C^+^)] primers 5104 and 5103 [designed to delete the replication origin and tetracycline resistance (Tc^R^) cassette of the plasmid pRG38 [89]] were used to amplify the kanamycin cassette of pKD3. The PCR product was electroporated into TH18710 (LT2/pKD46/pRG38) followed by selection for kanamycin resistance (Km^R^). Km^R^ colonies were pooled and infected with P22 to produce a transducing lysate. This lysate was used to transduce LT2 selecting Km^R^. The Km^R^ transductants were replica-plated in LB+Km and LB+Tc. Tc-sensitive (Tc^S^) and Km^R^ colonies should have resulted from integration of P*_flhDC_*-*luxCDBAE* into the chromosome generating a duplication of the promoter region of the *flhDC* operon. To check the integration of a single copy of P*_flhDC_*-*luxCDBAE*-Km and to screen for the presence of any duplication of the *luxCDBAE* upon integration, a set of primers [1401 (reverse for *luxC*)- 3091 (forward in upstream of Pwt*_flhDC_* promoter region not present in the plasmid pRG38)] demonstrated the correct integration of the plasmid at the *flhDC* promoter region. A second PCR reaction using [Primers 267 (Km) and 1403 (*luxE*)] demonstrated the correct placement of Km cassette after the luciferase operon. Amplification with primers, 1403 and 1401, indicated a single copy integration of the plasmid without its origin of replication. Five candidates were obtained having a single integration of Pwt*_flhDC_*-luciferase into the chromosome. One of the five candidates was sequenced and used in this study (TH18684). The Duplication of P*_flhDC_* was maintained in the presence of 5–10 µg/ml Km.

Mutations in the promoter region of P*_flhDC_*-*lux* were constructed using the λ-Red recombinase system, as reported previously [90], using the primers listed in Table S2. All transcriptional fusion constructs using the luciferase operon reporter used the strain TH18727: (DUP[(P*_flhDC_8093*::*tetRA*-luxCDBAE*Km*(P*_flhDC_flhD* ^+^ *flhC* ^+^)]/pKD46) as the electroporation recipient. Individual fusion constructs with specific promoter regions were designed as follows: the *rcsB* promoter region included 400 bp upstream of the start codon through 230 bp of coding region, the *rcsD* promoter region included 466 bp upstream of the start codon through 260 bp of coding region, the *slyA* promoter region included 258 bp upstream of the start codon and 290 bp of the coding region, the *hilD* promoter region included 300 bp upstream of the start codon through 240 bp of coding region, the *rtsA* promoter region included 264 bp upstream of the start codon through 290 bp of coding region, the *lrhA* promoter region included 880 bp upstream of the start codon through 200 bp of coding region and the *rflM* promoter region included 460 bp upstream of the start codon through 284 bp of coding region. The promoter regions defined above were amplified by PCR using the respective primers listed in Table S2, and electroporated into strain TH18727, using the Lambda-Red recombinase system selecting for replacement of *tetRA* element with a PCR-amplified DNA fragment [90].

### Construction of tagged proteins

Chromosomal FLAG-tagged HilD, RcsB and chromosomal HA-tagged RtsB, SlyA, RflM, HilD and LrhA were generated by the Lambda-Red recombinase system, as described previously [91] using gene-specific primer pairs, as shown in Table S2. All strains were verified by PCR amplification and DNA sequence analysis.

### Growth conditions and luciferase assays

LB+Km medium containing 1% tryptone, 0.5% yeast extract, and 0.5% NaCl was used for growth of all bacterial cultures to determine the transcription activities of luciferase. Overnight cultures in LB+Km cultures were adjusted to the same OD 595 nm, then, 8-ml glass tubes containing 2 ml of LB+Km were inoculated with a 500-fold dilution of the bacterial suspensions and incubated at 30°C in a water bath with shaking at 250 rpm. For determination of luciferase activity in batch cultures, samples (200 µl) were taken at different time point and the light production along with the OD595 were measured in 96 well plates in a microplate reader (PolarStar Optima). For the determination of luciferase activity in 96 well plates, adjusted OD595 of overnight bacterial cultures at 37°C were diluted 500-fold in LB+Km and 200 µl of diluted bacteria were added to 96 well dark plates (Greiner). The plates were sealed with breathe easy membrane (to minimize evaporation and to allow growth in semi-aerobic conditions) and incubated in a chamber/shaker of a PolarStar Optima microplate reader (BMG labtech) set at 30°C. The conditions of the plate reader to determine the light production and OD 595 nm were as follow: orbital Shaking for 300 s at 150 rpm, 5 s stop and 95 s for luciferase light reading of the wells. For normalization of results a 0.1 s integration time was used. The OD 595 nm and light production (luciferase) was measured over time using a PolarStar Optima microplate reader (BMG labtech). For the background, we took the average measurements of the strain (TH18402) harboring mutations in all the promoters of *flhDC*. After background correction, relative light units (Arbitrary Units) were calculated by dividing the lights reading with its corresponding OD 595 nm. The OD 595 nm in our setting of the PolarStar Optima reader corresponds to ∼1.69 factor of the OD 595 nm read with 1 ml spectrophotometer.

### Protein extraction and western blotting

Whole-cell extracts were prepared from samples of cultures grown in LB. 500-ml flasks containing 100 ml of LB were inoculated with a 500-fold dilution of the bacterial suspensions and incubated at 30°C in an orbital shaker at 150 rpm. Cells were collected at different optical densities (0.25, 0.4, 0.6, 0.8, 1 and 1.3) and washed twice with ice cold PBS. Pellets were lysed, at room temperature for 15 minutes, using B-PER reagent (Fisher, product #78243) with freshly added lysozyme (1 mg/ml) and protease inhibitors (Roche). The lysates were clarified by centrifugation at 4°C for 10 minutes. Supernatants were transferred to new eppendorfs and the extracted proteins were quantified using the BSA assay (BioRad). Samples, containing 50 µg of total protein per lane, were electrophoresed onto 12% to 14% Tris/SDS gels. To detect RtsA-HA a 15% Tricine-SDS gel was used as described [92]. Following transfer onto a 0.45 µm pore size polyvinylidene difluoride (PDVF) membrane (Immobilon P, Millipore) using a semidry transfer apparatus (Bio-Rad), membrane were blocked for 1 hour at room temperature with freshly prepared non-fat dry milk (5% w/v) in PBS. For detection of HA-tagged or Flag-tagged proteins, membrane blots were incubated overnight at 4°C with anti-HA (Covance) or anti-Flag M2 (Sigma) mouse monoclonal antibodies at 1∶1,000 and 1∶2,000 dilutions respectively. DnaK was detected using Anti-DnaK (Covance) diluted 1∶10,000. The blots were washed three times with PBS-T (PBS+0.1% tween) and incubated protected from light with green or red infrared dye-conjugated secondary antibody in non-fat dry milk (3% w/v) in PBS-T for 45 minutes at room temperature. Following three washes in PBS-T and one wash in PBS. Labeled proteins bands were detected using the Odyssey Infrared Imaging System (Li-COR Biosciences, Lincoln, NE, USA).

### Chromatin-Immunoprecipitation (CHIP)

CHIP was performed as in [93] with modifications. Bacterial batch cultures were grown at 30°C to different ODs, at which point formaldehyde (final concentration of 1%) was added to cells. After 20 min at room temperature in an orbital shaker, cross-linking was quenched by the addition of glycine (500 mM) for 10 minutes. Samples were then placed on ice for an additional 10 minutes to complete quenching. Cells were collected by centrifugation, and washed twice with cold phosphate-buffer saline (pH 7.5). Cells pellets were resuspended in 1 ml of lysis buffer (10 mM Tris, pH 8.0, 20% sucrose, 50 mM NaCl, 10 mM EDTA, 10 µg/ml of lysozyme) and incubated at 37°C for 30 min. Following lysis, 1 ml of immunoprecipitation buffer (50 mM HEPES-KOH, pH 7.5, 150 mM NaCl, 10 mM EDTA, 1% Triton X-100, 0.1% sodium deoxycholate, 0.1% sodium dodecyl sulfate) and phenylmethylsulfonyl fluoride (final concentration of 1 mM) were added. To shear cellular DNA to an average size of 500 to 1,000 bp, the cell extracts were sonicated on ice using Misonix Sonicator 3000 with a microtip at power 2 for three 10 s pulses, with 30 s rests on ice between pulses. The lysates were clarified by centrifugation and the supernatant were treated with 5 µl RNaseA (10 µg/ml) at 37°C for 30 minutes. The treated supernatant was retained for use as the input sample in the immunoprecipitation experiments. Aliquots of sheared samples were uncross-linked by incubation for 2 h at 42°C and 6 h at 65°C in 0.5× elution buffer containing freshly added 0.8 mg/ml of Proteinase K. DNA was purified using a PCR purification Kit (Bioline). An aliquot of purified DNA was run in a 1.25% agarose gel to confirm the shearing of DNA to 500–1000 bp fragments and DNA was quantified using Nanodrop spectrophotometer. An Aliquot of the input sample (2 µg) was used for each immunoprecipitation experiment. The sample was incubated with 50 µl of proteinPlus A/G beads (Santa Cruz) and 4 µl of HA monoclonal antibody (Covance) or Flag M2 antibody (Sigma) for 90 min at room temperature on a rotating wheel. An immunoprecipitation experiment without antibody was also set up as a negative control. The beads were collected by centrifugation and subsequently washed three time with immunoprecipitation buffer and once with immunoprecipitation buffer plus 300 mM NaCl, once with wash buffer (10 mM Tris-HCl, pH 8.0, 250 mM LiCl, 1 mM EDTA, 0.5% Nonidet-P40, 0.5% sodium deoxycholate) and finally with PBS buffer (pH 7.5). Immunoprecipitated complexes were then removed from the beads by treatment with elution buffer (50 mM Tris-HCl [pH 7.5], 10 mM EDTA, 1% SDS). Crosslinking of immunoprecipitated samples was reversed by incubation for 2 h at 42°C and 6 h at 65°C in 0.5× elution buffer with 0.8 mg/ml of Pronase (Roche). Prior to analysis, DNA was purified from the immunoprecipitate by using a PCR purification kit (Bioline) and resuspended in 30 µl of TE and quantified using a Nanodrop spectrophotometer. Two micrograms of the fragmented DNA, isolated from DNA-protein complexes, was used as the input in all ChIP assays. Following purification, Real-time PCRs were run on a C1000 thermal cycler (BioRad) to analyze immunoprecipitated DNA. DNA samples were used in a 20 µl reaction mix containing a 1 µM concentration of each oligonucleotide and 10 µl of 2× SYBR-Green Reaction mix. Two pairs of primers, 3569-3477 and 3753-3090 covering the promoter region of *flhDC* were used (Table S2). PCR conditions were as follow: Initial denaturation at 95°C for 3 min, and 40 cycles of 95°C for 15 s and 60°C for 1 min, followed by the default melting curve program of the PCR machine. Fold-enrichments were determined by the 2^−ΔCT^ method described in SA Biosciences User manual. To account for chromatin sample preparation differences, CHIP DNA fractions Ct values (Mean threshold cycles) were normalized (ΔCt(normalized ChIP) to the Input DNA fraction Ct values by substracting the Ct-values of the sample from the corresponding no antibody control. The percentage input of each ChIP fraction was calculated using 2^(−ΔCt(normalized ChIP)^ and adjusted to the normalized background (No antibody) using the following formula: ΔΔCt(Chip) = ΔCt(normalized ChIP)−ΔCt(normalized NoAb). The IP fold enrichment was then calculated using 2^(−ΔΔCt(ChIP/NAC))^ to evaluate the fold amount of starting material of the sample applied in the real-time PCR.

## Supporting Information

Figure S1 **Effect of static culture growth on the transcription of the** ***flhDC*** **operon in** ***Salmonella enterica*** **serovar Typhimurium.** This plot represents the luminescence divided by the corresponding OD595 (A.U.) of a static culture. An overnight culture of strain Pwt*_flhDC_* (TH18684) at 37°C was diluted 1 to 500 into fresh LB media. Cells were then incubated statically at 30°C and luminescence was recorded along with the OD595. OD values are shown at the bottom of the chart. Values are the average of two experiments done in duplicate.
(TIF)

Figure S2 **Transcription dynamics of factors that regulate** ***flhDC*** **transcription mimic the time in the cell growth phase where their effect on** ***flhDC*** **operon transcription is exerted.** Luciferase activity was investigated in wild-type strain harboring Pwt*_flhDC_-luxCDBAE-*Pwt*_flhDC_flhD^+^C^+^* (TH18684), *P_lrhA_-luxCDBAE* (TH20540), *P_rcsD_-luxCDBAE* (TH20087), *P_slyA_-luxCDBAE* (TH19426), *P_hilD_-luxCDBAE* (TH19425) *and P_rtsA_-luxCDBAE* (TH19664). Luciferase activity was recorded and plotted as described in Figure 2. (**A**) Transcription of the auto-regulated *lrhA* gene promoter was activated immediately after dilution of an overnight culture into LB media and earlier than the transcription of the *flhDC* operon. (**B**) The transcriptional profiles of *rflM* and *rcsD* promoters are shown in the second axis along with the Pwt*_flhDC_*. The activation of the *rflM* promoter transcription, expressed from an FlhD_4_C_2_-dependent promoter, was concomitant with that of *flhDC* operon transcription (Pwt*_flhDC_*), happening at earlier time point of the cell growth phase. The transcription from the *rflM* promoter (P*_rflM_*) diminished before cells enter stationary phase compared to promoter transcription for other regulator factors shown in this figure. Transcription from the *rcsD* promoter (P*_rcsD_*) (shown in the second axis) was activated before that of Pwt*_flhDC_*. (**C & D**) Activation of promoters of the virulence related genes, implicated in the regulation of *flhDC* transcription, took place after initiation of *flhDC* transcription. A representative growth curve is shown in (**A**, **C** & **D**). For (**B**), the OD595 is shown at the bottom of the chart.
(TIF)

Figure S3 **Analysis of mutations of the putative promoters P2, P3, P4 and P6. (A)** The wild-type sequence of −10 box of the putative TSSs and their mutant alleles are shown. (**B, C, D & E**) Charts represent the luciferase activities of the Pwt*_flhDC_-luxCDBAE-*Pwt*_flhDC_flhD^+^C^+^* reporter construct in wild-type and isogenic strains carrying mutations in individual start-site −10 boxes. Cells were grown overnight in LB and diluted 1 to 500 in fresh media, and grown at 30°C with shaking and luciferase activities were recorded at two optical densities (0.5, black bars and 1, grey bars). Charts of luciferase activity in strains with mutations in the P2 (**B**), P3 (**C**), P4 (**D**), P6 (**E**) promoters of *flhDC* operon compared to the wild-type *flhDC* promoter activity that was set at 100%. Each specific mutation is indicated under their corresponding bars. (**F**) Luciferase activity of strains P5^+^P6^+^P2^+^ (harboring mutations in P1, P3 and P4) and P6^+^P2^+^P1^+^ (harboring mutations in P5, P4 and P3). Results are the average of three independent experiments done in duplicate. Error bars represent standard deviation. (**G & H**) Mutations in the *flhDC* P2*_flhDC_* and P6*_flhDC_* promoter start-sites inhibit CRP-mediated transcriptional activation of P1*_flhDC_* start-site. (**G**) CRP does no longer affect transcription of *flhDC* in strains deficient in P1, P2 and P6 promoters. Luciferase activity of Pwt*_flhDC_*, P1^−^ *_flhDC_*, P2^−^ *_flhDC_* and P6^−^ *_flhDC_* was measured in two genetic backgrounds: wild-type (wt) and its isogenic null mutant *crp (crp*::Tn*10)*. Plots represent the ratio of the luciferase activity measured in wild-type strain relative to *crp* null mutant. (**H**) CRP represses transcription of P6*_flhDC_* promoter. Luciferase activity of P1^+^ *_flhDC_* (only P1 is active the rest of the promoters are mutated) and P6^+^ *_flhDC_* (only P6 is active the rest of the promoters are mutated) was measured in two genetic backgrounds: wild-type and its isogenic null mutant *crp*. Plots represent the ratio of the luciferase activity measured in *crp* null mutant relative to the wild-type.
(TIF)

Figure S4 **Effects of RcsB, LrhA, RtsB and SlyA regulators on** ***flhDC*** **P1** ***_flhDC_*** **and P5** ***_flhDC_*** **transcription.** For these assays, we compared the transcription of *flhDC* promoter region constructs (**A**) The P5^+^ *_flhDC_* (defective in P1, P2, P3, P4, and P6 start-sites) promoter constructs transcribed *flhDC* primarily from the P5 start-site. (**B**) The P1^+^ *_flhDC_* (defective in P2, P3, P4, P5 and P6 start-sites) promoter constructs transcribed *flhDC* primarily from the P1 start-site. (**A**) RcsB and LrhA but not RtsB or SlyA repressed transcription of *flhDC* in P1^+^ *_flhDC_* construct. Luciferase activity of P1^+^ *_flhDC_-luxCDBAE-* Pwt*_flhDC_flhD* ^+^ *C* ^+^ transcriptional fusion was investigated in five genetic backgrounds: wild-type (TH18901), Δ*rcsB*::*tetRA* (TH19217), *rtsB*::T-POP (TH19176), *lrhA*::T-POP (TH19603), *slyA*::T-POP (TH19618). (**B**) RcsB, LrhA and SlyA but not RtsB repressed transcription of *flhDC* in P5^+^ *_flhDC_* construct. Luciferase activity of P5^+^ *_flhDC_ -luxCDBAE-*P_wt_ *flhD* ^+^ *C* ^+^ transcriptional fusion was measured in wild-type (TH18905), Δ*rcsB*::*tetRA* (TH19221), *rtsB*::T-POP (TH19180), *lrhA::T-POP* (TH19607) and *slyA*::T-POP (TH19619). Plots represent luciferase activity divided by the OD595 and plotted against the OD595 values shown at the bottom of the chart.
(TIF)

Figure S5 **Effects of HA or Flag-tagged regulators, LrhA, RcsB, SlyA, HilD, RtsB and RflM on** ***flhDC*** **operon transcription. Luciferase activity of** Pwt*_flhDC_ -luxCDBAE-*P_wt_ *flhD* ^+^ *C* ^+^ transcriptional fusion was measured in wild-type, *lrhA*-HA, *rcsB*-Flag, *slyA*-HA, *hilD*-Flag, *rtsB*-HA and *rflM*-HA. Cells were diluted 1 to 500 from an overnight culture into LB and grown at 30°C. Plots represent luciferase activity measured at OD 1 compared to the wild-type expression set at 100%.
(TIF)

Table S1 **List of strains used in this study.**
(DOCX)

Table S2 **List of primers used in this study.**
(DOCX)

Text S1 **Analysis of the promoters of the** ***flhDC*** **operon.**
(DOCX)
