# Supplementary figures and images for: The Effect of Cell Growth Phase on the Regulatory Cross-Talk between Flagellar and Spi1 Virulence Gene Expression
Source: PLoS Pathog. 2014 Mar 6;10(3):e1003987. doi: 10.1371/journal.ppat.1003987 (PMC3946378; doi:10.1371/journal.ppat.1003987)

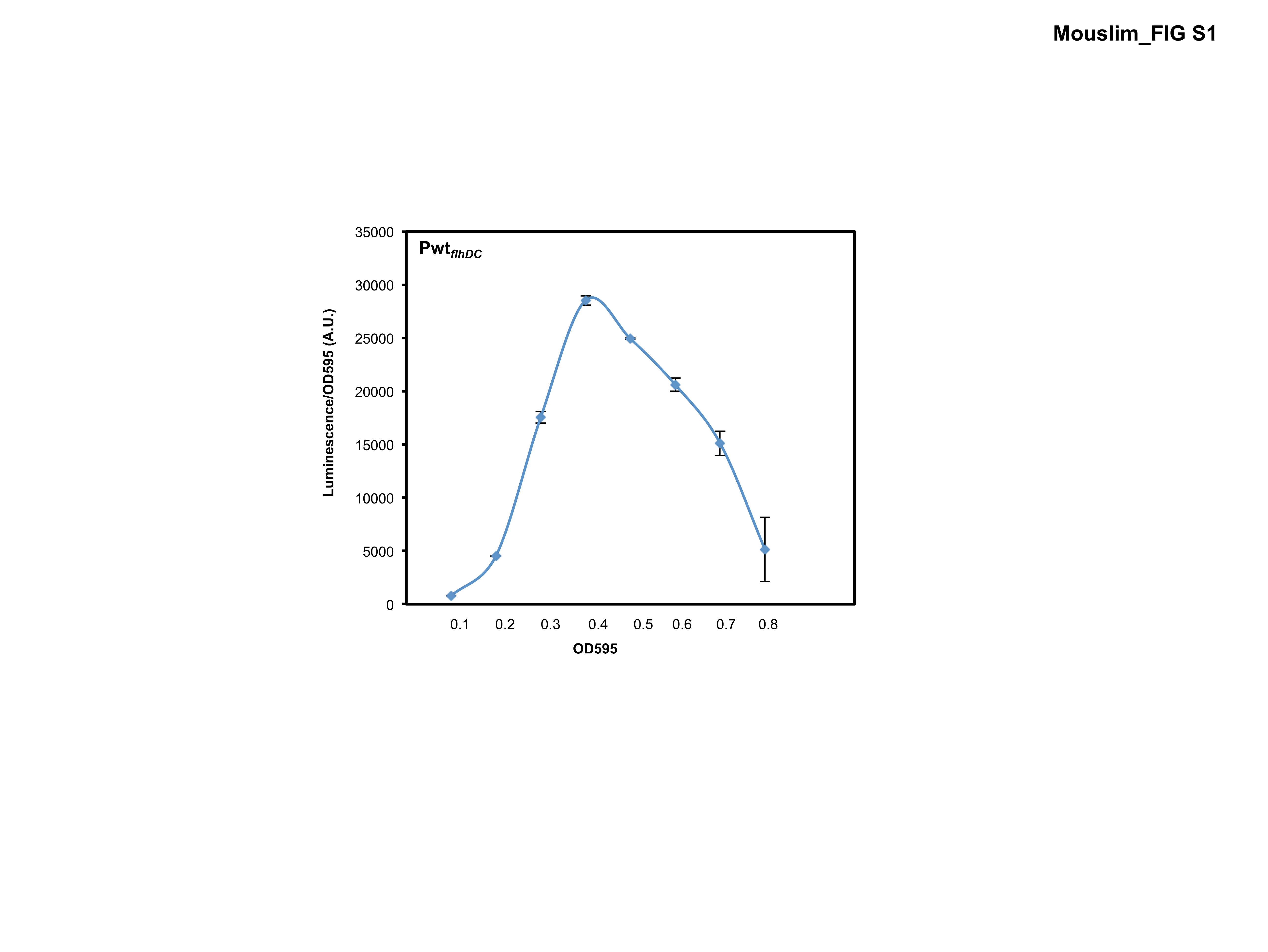

Supplement: Figure S1 — Effect of static culture growth on the transcription of the flhDC operon in Salmonella enterica serovar Typhimurium. This plot represents the luminescence divided by the corresponding OD595 (A.U.) of a static culture. An overnight culture of strain PwtflhDC (TH18684) at 37°C was diluted 1 to 500 into fresh LB media. Cells were then incubated statically at 30°C and luminescence was recorded along with the OD595. OD values are shown at the bottom of the chart. Values are the average of two experiments done in duplicate. (TIF) [file ppat.1003987.s001.tif]

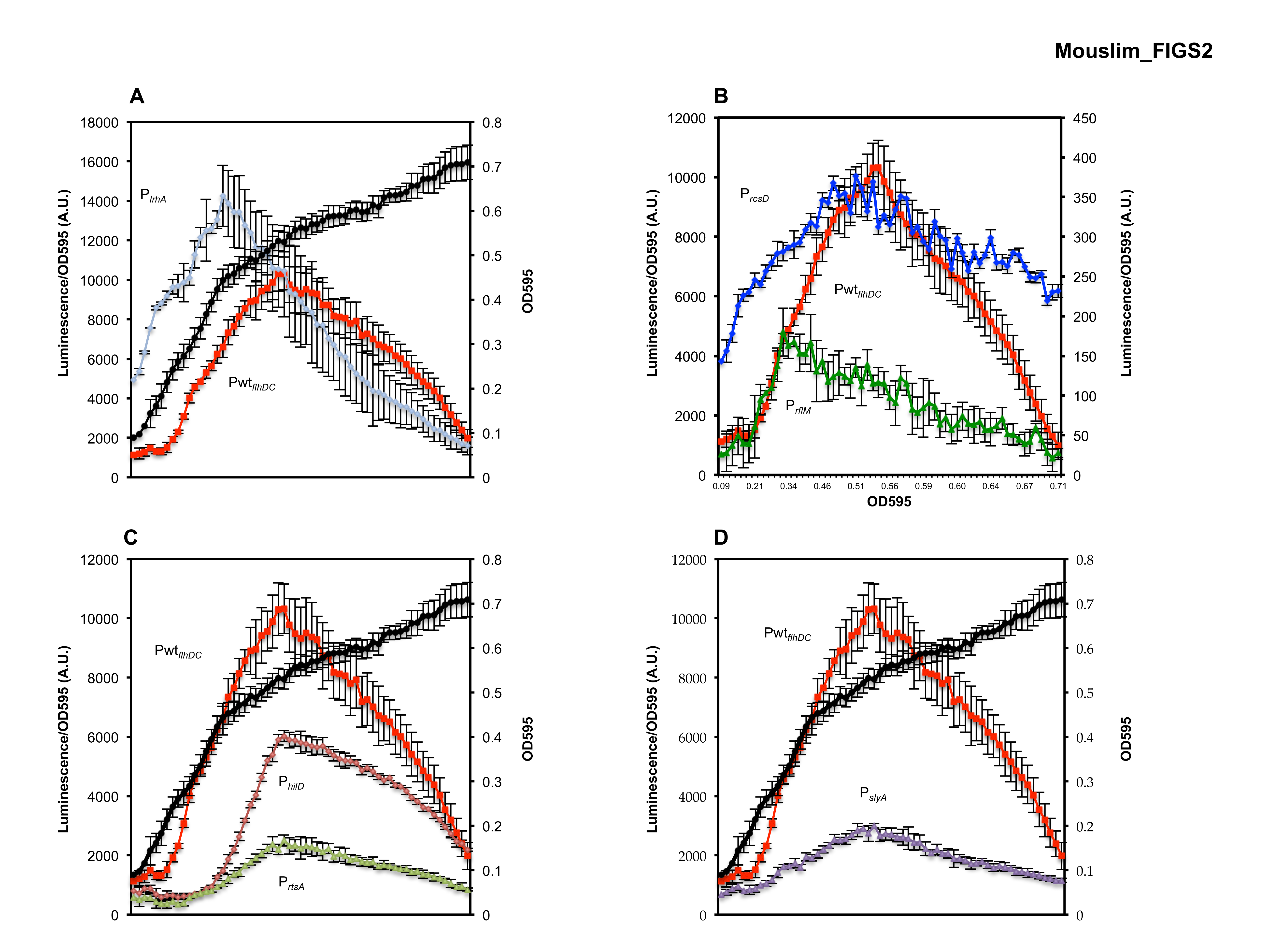

Supplement: Figure S2 — Transcription dynamics of factors that regulate flhDC transcription mimic the time in the cell growth phase where their effect on flhDC operon transcription is exerted. Luciferase activity was investigated in wild-type strain harboring PwtflhDC-luxCDBAE-PwtflhDCflhD+C+ (TH18684), PlrhA-luxCDBAE (TH20540), PrcsD-luxCDBAE (TH20087), PslyA-luxCDBAE (TH19426), PhilD-luxCDBAE (TH19425) and PrtsA-luxCDBAE (TH19664). Luciferase activity was recorded and plotted as described in Figure 2. (A) Transcription of the auto-regulated lrhA gene promoter was activated immediately after dilution of an overnight culture into LB media and earlier than the transcription of the flhDC operon. (B) The transcriptional profiles of rflM and rcsD promoters are shown in the second axis along with the PwtflhDC. The activation of the rflM promoter transcription, expressed from an FlhD4C2-dependent promoter, was concomitant with that of flhDC operon transcription (PwtflhDC), happening at earlier time point of the cell growth phase. The transcription from the rflM promoter (PrflM) diminished before cells enter stationary phase compared to promoter transcription for other regulator factors shown in this figure. Transcription from the rcsD promoter (PrcsD) (shown in the second axis) was activated before that of PwtflhDC. (C & D) Activation of promoters of the virulence related genes, implicated in the regulation of flhDC transcription, took place after initiation of flhDC transcription. A representative growth curve is shown in (A, C & D). For (B), the OD595 is shown at the bottom of the chart. (TIF) [file ppat.1003987.s002.tif]

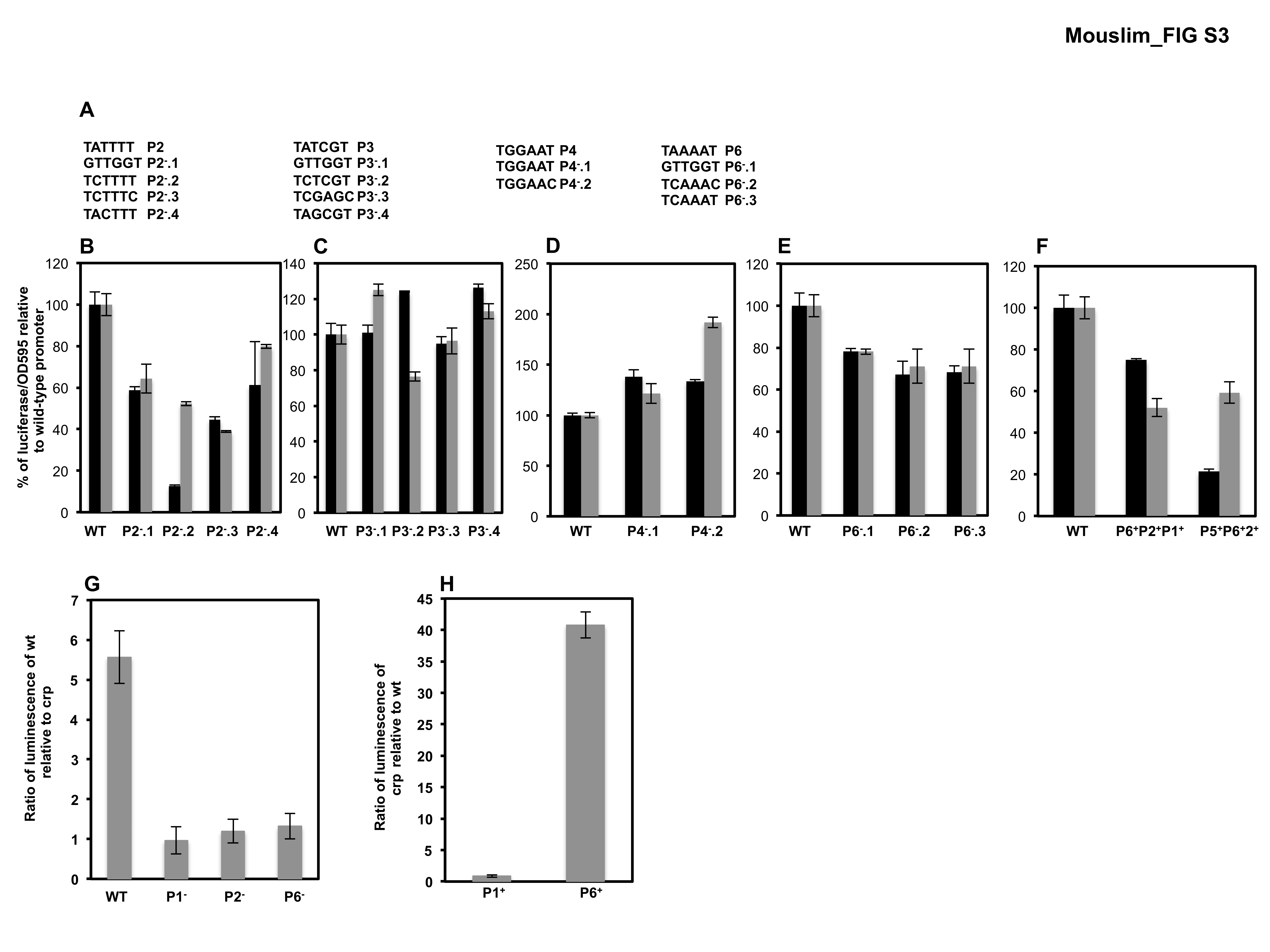

Supplement: Figure S3 — Analysis of mutations of the putative promoters P2, P3, P4 and P6. (A) The wild-type sequence of −10 box of the putative TSSs and their mutant alleles are shown. (B, C, D & E) Charts represent the luciferase activities of the PwtflhDC-luxCDBAE-PwtflhDCflhD+C+ reporter construct in wild-type and isogenic strains carrying mutations in individual start-site −10 boxes. Cells were grown overnight in LB and diluted 1 to 500 in fresh media, and grown at 30°C with shaking and luciferase activities were recorded at two optical densities (0.5, black bars and 1, grey bars). Charts of luciferase activity in strains with mutations in the P2 (B), P3 (C), P4 (D), P6 (E) promoters of flhDC operon compared to the wild-type flhDC promoter activity that was set at 100%. Each specific mutation is indicated under their corresponding bars. (F) Luciferase activity of strains P5+P6+P2+ (harboring mutations in P1, P3 and P4) and P6+P2+P1+ (harboring mutations in P5, P4 and P3). Results are the average of three independent experiments done in duplicate. Error bars represent standard deviation. (G & H) Mutations in the flhDC P2flhDC and P6flhDC promoter start-sites inhibit CRP-mediated transcriptional activation of P1flhDC start-site. (G) CRP does no longer affect transcription of flhDC in strains deficient in P1, P2 and P6 promoters. Luciferase activity of PwtflhDC, P1− flhDC, P2− flhDC and P6− flhDC was measured in two genetic backgrounds: wild-type (wt) and its isogenic null mutant crp (crp::Tn10). Plots represent the ratio of the luciferase activity measured in wild-type strain relative to crp null mutant. (H) CRP represses transcription of P6flhDC promoter. Luciferase activity of P1+ flhDC (only P1 is active the rest of the promoters are mutated) and P6+ flhDC (only P6 is active the rest of the promoters are mutated) was measured in two genetic backgrounds: wild-type and its isogenic null mutant crp. Plots represent the ratio of the luciferase activity measured in crp null mutant relativ [file ppat.1003987.s003.tif]

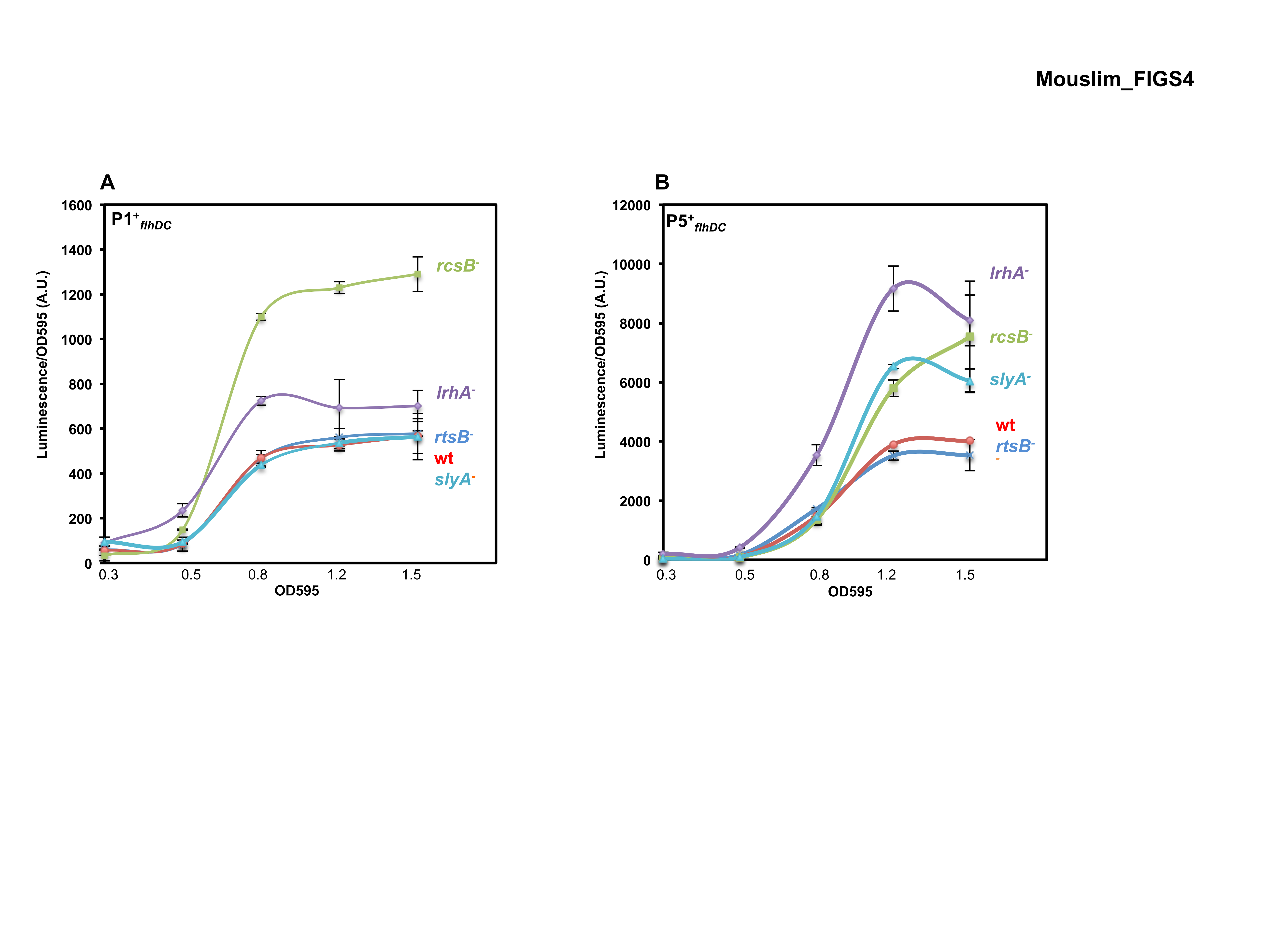

Supplement: Figure S4 — Effects of RcsB, LrhA, RtsB and SlyA regulators on flhDC P1 flhDC and P5 flhDC transcription. For these assays, we compared the transcription of flhDC promoter region constructs (A) The P5+ flhDC (defective in P1, P2, P3, P4, and P6 start-sites) promoter constructs transcribed flhDC primarily from the P5 start-site. (B) The P1+ flhDC (defective in P2, P3, P4, P5 and P6 start-sites) promoter constructs transcribed flhDC primarily from the P1 start-site. (A) RcsB and LrhA but not RtsB or SlyA repressed transcription of flhDC in P1+ flhDC construct. Luciferase activity of P1+ flhDC-luxCDBAE- PwtflhDCflhD + C + transcriptional fusion was investigated in five genetic backgrounds: wild-type (TH18901), ΔrcsB::tetRA (TH19217), rtsB::T-POP (TH19176), lrhA::T-POP (TH19603), slyA::T-POP (TH19618). (B) RcsB, LrhA and SlyA but not RtsB repressed transcription of flhDC in P5+ flhDC construct. Luciferase activity of P5+ flhDC -luxCDBAE-Pwt flhD + C + transcriptional fusion was measured in wild-type (TH18905), ΔrcsB::tetRA (TH19221), rtsB::T-POP (TH19180), lrhA::T-POP (TH19607) and slyA::T-POP (TH19619). Plots represent luciferase activity divided by the OD595 and plotted against the OD595 values shown at the bottom of the chart. (TIF) [file ppat.1003987.s004.tif]
