# Supplementary material for: The Effect of Cell Growth Phase on the Regulatory Cross-Talk between Flagellar and Spi1 Virulence Gene Expression
Source: PLoS Pathog. 2014 Mar 6;10(3):e1003987. doi: 10.1371/journal.ppat.1003987 (PMC3946378; doi:10.1371/journal.ppat.1003987)
Supplement: Table S1 — List of strains used in this study. (DOCX) [file ppat.1003987.s006.docx]

**Table S1. List of strains used in this study**

Strains Genotypes Origin

TH407 LT2 John Roth

TH12891 Δ*rflM5*::FCF Lab collection

TH15627 *lrhA*::T-POP Lab collection

TH15937 *rtsB*::T-POP Lab collection

TH15634 *slyA*::T-POP Lab collection

TH9330 ∆*rcsB*::*tetRA* Lab collection

TH18684 DUP[(P*_flhD_*_C_*8093*)**luxCDABE*-Km*-(P*flhD^+^C*^+^)]

TH18710 LT2/pKD46/pRG38 (P*flhDC*-*luxCDBAE* TcR)

TH18855 DUP[(P*_flhD_*_C_*8118*)**luxCDABE*-Km*-(P*flhD^+^C^+^*)] P1^-^.1

TH18856 DUP[(P*_flhD_*_C_*8119*)**luxCDABE*-Km*-(P*flhD^+^C^+^*)] P2^-^.1

TH18857 DUP[(P*_flhD_*_C_*8120*)**luxCDABE*-Km*-(P*flhD^+^C^+^*)] P3^-^.1

TH18858 DUP[(P*_flhD_*_C_*8121*)**luxCDABE*-Km*-(P*flhD^+^C^+^*)] P4^-^.1

TH18859 DUP[(P*_flhD_*_C_*8122*)**luxCDABE*-Km*-(P*flhD^+^C^+^*)] P5^-^.1

TH18860 DUP[(P*_flhD_*_C_*8123*)**luxCDABE*-Km*-(P*flhD^+^C^+^*)] P6^-^.1

TH18888 DUP[(P*_flhD_*_C_*8109*)**luxCDABE*-Km*-(P*flhD^+^C^+^*)] P1^-^.2

TH18889 DUP[(P*_flhD_*_C_*8110*)**luxCDABE*-Km*-(P*flhD^+^C^+^*)] P1^-^.3

TH20533 DUP[(P*_flhD_*_C_*8230*)**luxCDABE*-Km*-(P*flhD^+^C^+^*)] P1^-^.4

TH18889 DUP[(P*_flhD_*_C_*8111*)**luxCDABE*-Km*-(P*flhD^+^C^+^*)] P2^-^.1

TH18891 DUP[(P*_flhD_*_C_*8112*)**luxCDABE*-Km*-(P*flhD^+^C^+^*)] P2^-^.2

TH20538 DUP[(P*_flhD_*_C_*8235*)**luxCDABE*-Km*-(P*flhD^+^C^+^*)] P2^-^.3

TH18892 DUP[(P*_flhD_*_C_*8113*)**luxCDABE*-Km*-(P*flhD^+^C^+^*)] P3^-^.1

TH18993 DUP[(P*_flhD_*_C_*8114*)**luxCDABE*-Km*-(P*flhD^+^C^+^*)] P3^-^.2

TH20537 DUP[(P*_flhD_*_C_*8234*)**luxCDABE*-Km*-(P*flhD^+^C^+^*)] P3^-^.3

TH18894 DUP[(P*_flhD_*_C_*8115*)**luxCDABE*-Km*-(P*flhD^+^C^+^*)] P4^-^.2

TH18895 DUP[(P*_flhD_*_C_*8116*)**luxCDABE*-Km*-(P*flhD^+^C^+^*)] P5^-^.2

TH18896 DUP[(P*_flhD_*_C_*8117*)**luxCDABE*-Km*-(P*flhD^+^C^+^*)] P5^-^.3

TH20536 DUP[(P*_flhD_*_C_*8233*)**luxCDABE*-Km*-(P*flhD^+^C^+^*)] P5^-^.4

TH20534 DUP[(P*_flhD_*_C_*8231*)**luxCDABE*-Km*-(P*flhD^+^C^+^*)] P6^-^.1

TH20535 DUP[(P*_flhD_*_C_*8232*)**luxCDABE*-Km*-(P*flhD^+^C^+^*)] P6^-^.2

TH18901 DUP[(P*_flhD_*_C_*8124*)**luxCDABE*-Km*-(P*flhD^+^C^+^*)] P1^+^

TH18902 DUP[(P*_flhD_*_C_*8125*)**luxCDABE*-Km*-(P*flhD^+^C^+^*)] P2^+^

TH18903 DUP[(P*_flhD_*_C_*8126*)**luxCDABE*-Km*-(P*flhD^+^C^+^*)] P3^+^

TH18904 DUP[(P*_flhD_*_C_*8127*)**luxCDABE*-Km*-(P*flhD^+^C^+^*)] P4^+^

TH18905 Dup[(P*_flhD_*_C_*8128*)**luxCDABE*-Km*-(P*flhD^+^C^+^*)] P5^+^

TH18906 DUP[(P*_flhD_*_C_*8129*)**luxCDABE*-Km*-(P*flhD^+^C^+^*)] P6^+^

TH18913 DUP[(P*_flhD_*_C_*8136*)**luxCDABE*-Km*-(P*flhD^+^C^+^*)] AP’s

TH20114 DUP[(P*_flhDC_8195*)**luxCDBAE*-Km*(P*flhD^+^C^+^*)] P5.1^-^ P1.3^-^

TH19860 DUP[(P*_flhDC_8173*)**luxCDBAE*-Km*(P*flhD^+^C^+^*)] P6^+^P2^+^P1^+^

TH19861 DUP[(P*_flhDC_8174*)**luxCDBAE*-Km*(P*flhD^+^C^+^*)] P5^+^P6^+^P2^+^

TH18716 DUP[(P*_flhD_*_C_*8093*)**luxCDABE*-Km*-(P*flhD^+^C^+^*)] ∆*rflM5*::FCF

TH18720 DUP[(P*_flhD_*_C_*8093*)**luxCDABE*-Km*-(P*flhD^+^C^+^*)] *slyA*::T-POP

TH18722 DUP[(P*_flhD_*_C_*8093*)**luxCDABE*-Km*-(P*flhD^+^C^+^*)] *lrhA*::T-POP

TH18724 DUP[(P*_flhD_*_C_*8093*)**luxCDABE*-Km*-(P*flhD^+^C^+^*)] *rtsB*::T-POP

TH19654 DUP[(P*_flhD_*_C_*8093*)**luxCDABE*-Km*-(P*flhD^+^C^+^*)] ∆*hilD221*::*tetRA*

TH19230 DUP[(P*_flhD_*_C_*8093*)**luxCDABE*-Km*-(P*flhD^+^C^+^*)] ∆*rcsB*::*tetRA*

TH20545 DUP[(P*_flhD_*_C_*8093*)**luxCDABE*-Km*-(P*flhD^+^C^+^*)] *∆araBAD938*::FCF

TH20546 DUP[(P*_flhD_*_C_*8093*)**luxCDABE*-Km*-(P*flhD^+^C^+^*)] *∆araBAD921*::*rflM*^+^

TH19961 DUP[(P*_flhD_*_C_*8110*)**luxCDABE*-Km*-(P*flhD^+^C^+^*)] ∆*rflM5*::FCF

TH19965 DUP[(P*_flhD_*_C_*8110*)**luxCDABE*-Km*-(P*flhD^+^C^+^*)] ∆*hilD221*::*tetRA*

TH19970 DUP[(P*_flhD_*_C_*8110*)**luxCDABE*-Km*-(P*flhD^+^C^+^*)] ∆*lrhA*::*tetRA*

TH19971 DUP[(P*_flhD_*_C_*8110*)**luxCDABE*-Km*-(P*flhD^+^C^+^*)] *slyA*::T-POP

TH19972 DUP[(P*_flhD_*_C_*8110*)**luxCDABE*-Km*-(P*flhD^+^C^+^*)] *rtsB*::T-POP

TH20236 DUP[(P*_flhD_*_C_*8110*)**luxCDABE*-Km*-(P*flhD^+^C^+^*)] ∆*rcsB*::*tetRA*

TH20007 DUP[(P*_flhD_*_C_*8110*)**luxCDABE*-Km*-(P*flhD^+^C^+^*)] ∆*araBAD938*::FCF

TH20009 DUP[(P*_flhD_*_C_*8110*)**luxCDABE*-Km*-(P*flhD^+^C^+^*)] ∆*araBAD921*::*rflM*^+^

TH19962 DUP[(P*_flhD_*_C_*8116*)**luxCDABE*-Km*-(P*flhD^+^C^+^*)] ∆*rflM5*::FCF

TH19966 DUP[(P*_flhD_*_C_*8116*)**luxCDABE*-Km*-(P*flhD^+^C^+^*)] ∆*hilD221*::*tetRA*

TH19974 DUP[(P*_flhD_*_C_*8116*)**luxCDABE*-Km*-(P*flhD^+^C^+^*)] ∆*lrhA*::*tetRA*

TH19975 DUP[(P*_flhD_*_C_*8116*)**luxCDABE*-Km*-(P*flhD^+^C^+^*)] *slyA*::T-POP

TH19976 DUP[(P*_flhD_*_C_*8116*)**luxCDABE*-Km*-(P*flhD^+^C^+^*)] *rtsB*::T-POP

TH20012 DUP[(P*_flhD_*_C_*8116*)**luxCDABE*-Km*-(P*flhD^+^C^+^*)] ∆*araBAD938*::FCF

TH20014 DUP[(P*_flhD_*_C_*8116*)**luxCDABE*-Km*-(P*flhD^+^C^+^*)] ∆*araBAD921*::*rflM*^+^

TH20237 DUP[(P*_flhD_*_C_*8116*)**luxCDABE*-Km*-(P*flhD^+^C^+^*)] ∆*rcsB*::*tetra*

TH19176 DUP[(P*_flhD_*_C_*8124*)**luxCDABE*-Km*-(P*flhD^+^C^+^*)] *rtsB*::T-POP

TH19217 DUP[(P*_flhD_*_C_*8124*)**luxCDABE*-Km*-(P*flhD^+^C^+^*)] ∆*rcsB*::*tetRA*

TH19585 DUP[(P*_flhD_*_C_*8124*)**luxCDABE*-Km*-(P*flhD^+^C^+^*)] ∆*rflM5*::FCF

TH19603 DUP[(P*_flhD_*_C_*8124*)**luxCDABE*-Km*-(P*flhD^+^C^+^*)] *lrhA*::T-POP

TH19618 DUP[(P*_flhD_*_C_*8124*)**luxCDABE*-Km*-(P*flhD^+^C^+^*)] *slyA*::T-POP

TH19180 DUP[(P*_flhD_*_C_*8128*)**luxCDABE*-Km*-(P*flhD^+^C^+^*)] *rtsB*::T-POP

TH19221 DUP[(P*_flhD_*_C_*8128*)**luxCDABE*-Km*-(P*flhD^+^C^+^*)] ∆*rcsB*::*tetRA*

TH19589 DUP[(P*_flhD_*_C_*8128*)**luxCDABE*-Km*-(P*flhD^+^C^+^*)] ∆*rflM5*::FCF

TH19607 DUP[(P*_flhD_*_C_*8128*)**luxCDABE*-Km*-(P*flhD^+^C^+^*)] *lrhA*::T-POP

TH19619 DUP[(P*_flhD_*_C_*8128*)**luxCDABE*-Km*-(P*flhD^+^C^+^*)] *slyA*::T-POP

TH19424 ∆*hilD221*::*tetRA*

TH19664 P*_rtsA_*-*113*-*luxCDBAE*-Km

TH19426 P*_slyA_*-*luxCDBAE*-Km

TH20540 P*_lrhA_*-*luxCDBAE*-Km

TH19736 P*_rflM_8*-*luxCDBAE*-Km

TH19425 P*_hilD_222*-*luxCDBAE*-Km

TH20087 P*_rcsB_*-*luxCDBAE*-Km

TH20539 P*_rcsD_*-*luxCDBAE*-Km

TH19687 P*_hilD_22*-*luxCDBAE*-Km *rcsC*::T-POP

TH19690 P*_hilD_22*-*luxCDBAE*-Km Δ*fliZ5738*::FCF

TH20541 P*_hilD_22*-*luxCDBAE*-Km Δ*938*::FCF

TH20542 P*_hilD_22*-*luxCDBAE*-Km Δ*921*::*rflM*^+^

TH19733 P*_hiD_222***luxCDBAE*-Km* *flhC5706*::Tn10*d*Cm

TH20543 P*_hilD_22*-*luxCDBAE*-Km Δ*938*::FCF P*_flhDC_5451*::T-POP

TH20544 P*_hilD_22*-*luxCDBAE*-Km Δ*921*::*rflM^+^* P*_flhDC_5451*::T-POP

TH19853 *rflM9*-2xHA-FCF

TH18629 *lrhA*-2xHA-FCF

TH20430 HA-*lrhA*

TH19855 *slyA*-2xHA-FCF

TH19854 *rtsB115*-2xHA-FCF

TH18628 *rcsB*-3xFlag-Km

TH20429 *hilD227*-3xFlag-Km

TH20415 *hilD228*-2xHA-FCF

TH19210 DUP[(P*_flhD_*_C_*8093*)**luxCDABE*-Km*-(P*flhD^+^C^+^*)] *crp-773*::Tn*10*

TH19191 DUP[(P*_flhD_*_C_*8118*)**luxCDABE*-Km*-(P*flhD^+^C^+^*)] *crp-773*::Tn*10*

TH19192 DUP[(P*_flhD_*_C_*8119*)**luxCDABE*-Km*-(P*flhD^+^C^+^*)] *crp-773*::Tn*10*

TH19196 DUP[(P*_flhD_*_C_*8123*)**luxCDABE*-Km*-(P*flhD^+^C^+^*)] *crp*-*773*::Tn*10*

TH19197 DUP[(P*_flhD_*_C_*8124*)**luxCDABE*-Km*-(P*flhD^+^C^+^*)] *crp*-*773*::Tn*10*

TH19202 DUP[(P*_flhD_*_C_*8129*)**luxCDABE*-Km*-(P*flhD^+^C^+^*)] *crp*-*773*::Tn*10*

TH19961 DUP[(P*_flhDC_8110*)**luxCDBAE*-Km*(P*flhD^+^C^+^*)] Δ*rflM5*::FCF

TH18726 DUP[(P*_flhDC_8095*::*tetRA***luxCDBAE*-Km*(P*flhD^+^C^+^*)]

TH18727 pKD46/ DUP[(P*_flhDC_8095*::*tetRA***luxCDBAE*-Km*(P*flhD^+^C^+^*)]

TH20119 P*_flhDC_8200* P5^-^P1^-^

TH18116 P*_flhDC_8043* P1^-^

TH18117 P*_flhDC_8044* P2^-^

TH18118 P*_flhDC_8045* P3^-^

TH18119 P*_flhDC_8046* P4^-^

TH18120 P*_flhDC_8047* P5^-^

TH18121 P*_flhDC_8048* P6^-^

All strains listed were originated in this study unless marked otherwise
