# Supplementary material for: The Effect of Cell Growth Phase on the Regulatory Cross-Talk between Flagellar and Spi1 Virulence Gene Expression
Source: PLoS Pathog. 2014 Mar 6;10(3):e1003987. doi: 10.1371/journal.ppat.1003987 (PMC3946378; doi:10.1371/journal.ppat.1003987)
Supplement: Table S2 — List of primers used in this study. (DOCX) [file ppat.1003987.s007.docx]

**Table S2. List of primers used in this study**

**Primers Primers DNA Sequence 5’3’**

**Number Name**

**Primers used to verify the integration of P*_flhDC_*-*luxCDBAE* into the chromosome**

267 K1 CAGTCATAGCCGAATAGCCT

268 K2 CGGTGCCCTGAATGAACTGC

5104 ΔP155APRG38 CTGACGGGGTGGTGCGTAACGGCAAAAGCACCGCCGGACGTGTAGGCTGGAGCTGCTTCG

5105 ΔTETPRG38 GAGTGGTGAATCCGTTAGCGAGGTGCCGCCGGCTTCCATCATATGAATATCCTCCTTAG

1401 luxC.R CGGGAAAGATTTCAACCTGGC

1403 luxE.R TCGCGTCAATACGAGGACG

545 *flhD*.R GCGGTTGCGGACACTGCTC

3091 P*flhDC*.F ATTTCTGCAATGCGCTAATGC

**Primers used for construction of transcriptional fusion to regulators of *flhDC***

5542 *hilD*-LUX.R GAAATTTTTTTAGTCATACGTATCCTCCAAGCCTGAATTGTTTGAGCACCAACATCCC

5541 *hilD*-LUXF CATTTCTGCAATGCGCTAATGCCACATTAATGTGAAGGAATATACTGTTAGCGATGTC

5549 *slyA*-LUXF CATTTCTGCAATGCGCTAATGCCACATTAATGTGAAGGAAAGCAATGTTCCTTTGCG

5550 *slyA*-LUXR GAAATTTTTTTAGTCATACGTATCCTCCAAGCCTGAATTGCTGCTCAATGCCTATCGC

5546 *rflM*-LUXR GAAATTTTTTTAGTCATACGTATCCTCCAAGCCTGAATTGTGGCATATTGCGGTCTG

5585 *rflM*-LUXF ATTTCTGCAATGCGCTAATGCCACATTAATGTGAAGGAAACGGCGCCGATGCCGGTC

5543 *rtsA*-LUXF ATTTCTGCAATGCGCTAATGCCACATTAATGTGAAGGATTTTAAAGAGATAAATTG

5544 *rtsA*-LUXR GAAATTTTTTTAGTCATACGTATCCTCCAAGCCTGAATTGGACGCTGAATCTAATTC

5586 *lrhA*-LUXF CATTTCTGCAATGCGCTAATGCCACATTAATGTGAAGGACGAAACAATGTACTCCCACG

5548 *lrhA*-LUXR GAAATTTTTTTAGTCATACGTATCCTCCAAGCCTGAATTGAAAAGTTTGTTACGGCCATG

6037 *rcsB*-LUXR GAAATTTTTTTAGTCATACGTATCCTCCAAGCCTGAATTCGGCAGGTTGTTGATCAATGC

6072 *rcsB*-LUXF CATTTCTGCAATGCGCTAATGCCACATTAATGTGAAGGATGAGCAATGCTATGCAGG

6074 *rcsD*-LUXF CATTTCTGCAATGCGCTAATGCCACATTAATGTGAAGGAGATGAAATTCATAGCACTG

6075 *rcsD*-LUXR GAAATTTTTTTAGTCATACGTATCCTCCAAGCCTGAATTCCTGTCGATGGCGTAGTGGTC

**Primers used for construction of in-frame fusion to regulators of *flhDC***

5086 *rcsB*-FLAG-F CTCTCTTCTGTCACCCTGAGTCCGACAGACAAAGAAGACTACAAAGACCATGACGG

5087 *rcsB*-FLAG-R GCGTAGCGCCATCAGGCTGGGTAACATAAAACGCATCATATGAATATCCTCCTTAG

5937 *rtsB*-HA.F GCCTTGCCTACCACTCTACCAACATTTTAGGAAAAATTACATATGAATATCCTCCTTAG

5636 *rtsB*-HA.F TTGATGCTGAATGAGTTTTACATATCAGTCGATATTACGTATCCGTATGATGTTCCTGAT

5930 *rflM*-HA.F CGGTATTCTCCCTTTCTTATGCTTCCGGAGAAAATCGATTATCCGTATGATGTTCCTGAT

5931 *rflM*-HA.R CAGTAATCATCAACGGTACGGCATGGCGTCGTACCGTAACATATGAATATCCTCCTTAG

5933 *slyA*-HA.R CGTGTGGTCACATGGCCACACGTATGCCCCTGCACCTCACATATGAATATCCTCCTTAG

5932 *slyA*-HA.F GCCCTTGAACACAATATTATGGAATTGCACTCTCACGATTATCCGTATGATGTTCCTGAT

5088 *lrhA*-HA.F GGAGATGATCCGCTGATGGTGGAAGGGGGTTTTGAGTATCCGTATGATGTTCCTGAT

5102 *lrhA*-HA.R CTTTTTTACATTTACGACTTAGCGCCTTTTGCCTGCGAGCATATGAATATCCTCCTTAG

6145 HA-*lrhA*1 GTCAGCCCGATATGACCCGCCAGTAAGTGAAAAATTATGTATCCGTATGATGTTCCTGAT

6146 HA-*lrhA*2 TATCCGTATGATGTTCCTGATTATGCTAGCCTCATAAATGCAAATCGTCCGATAATTAAC

6127 *hilD*-FLAG-F AAAACTACGCCATCGACATTCATAAAAATGGCGAACCATGACTACAAAGACCATGACGG

6126 *hilD*-FLAG.R TAAAAATCTTTACTTAAGTGACAGATACAAAAAATGCATATGAATATCCTCCTTAG

5935 *hilD*-HA.F TAAAAATCTTTACTTAAGTGACAGATACAAAAAATGTTACATATGAATATCCTCCTTAG

**Primers used in RT-PCR**

3090 P*flhDC*.F GGAATACTTACGATAAAACCATCAGC

3477 P*flhDC*.R GTTGTATGTCACGAAGCTG

3569 P*flhDC*.F CCACTGAGATTCGCCTTACAC

3753 P*flhDC*.F TAAATGGGTGAACAAGGAAAGC

**Primers used for deletion of *hilD***

5533 Δ*hilD-*TetR AAATAACATCAACAAAGGGATAATATGGAAAATGTAACCTTAAGACCCACTTTCACATT

5534 Δ*hilD-*TETA CTTAAGTGACAGATACAAAAAATGTTAATGGTTCGCCATCTAAGCACTTGTCTCCTG

**Primers used for construction of P*flhDC* mutations**

4961 P1.R TTTACAAATGCCTAAGATTTTTCCTAATTCGACGCAACCAACCTCGTCAGCTTCGTGACA

5200 P1.1 TGTCACGAAGCTGACGAGTCGAGCTGCGTCGAATTAGGAAAAATCTTAGGCATTTGTAAA

5201 P1.2 TTTACAAATGCCTAAGATTTTTCCTAATTCGACGCAGCTCGACTCGTCAGCTTCGTGACA

5202 P1.3 TTTACAAATGCCTAAGATTTTTCCTAATTCGACGCAACTCGACTCGTCAGCTTCGTGACA

5440 P1.4 TTTACAAATGCCTAAGATTTTTCCTAATTCGACGCAACCCTACTCGTCAGCTTCGTGACA

5198 P2.1 GTAAGTATTCCGTTAAAATATGTGATCTGCATCACATCTTTCCTAAAATCGCCGTCCCGC

5199 P2.2 GTAAGTATTCCGTTAAAATATGTGATCTGCATCACATCTTTTCTAAAATCGCCGTCCCGC

5441 P2.3 GTAAGTATTCCGTTAAAATATGTGATCTGCATCACATATCTTCTAAAATCGCCGTCCCGC

5094 P3.1.F ATTAATTAAACAAAGTAAAAGCCATGCTGATGGTTTTCTCGCAAGTATTCCGTTAAAATA

5587 P3.1.R TATTTTAACGGAATACTTGCGAGAAAACCATCATGGCTTTTAC

5195 P3.2 ATTAATTAAACAAAGTAAAAGCCATGCTGATGGTTTTCTCGTAAGTATTCCGTTAAAATA

5442 P3.3 ATTAATTAAACAAAGCCATGCTGATGGTTTTACCGTAAGTATTCCGTTAAAATA

4958 P3.R ACGGAATACTTACCAACAAACCATCAGCATGGCTTTTAC

4960 P3F TGGTTTGTTGGTAAGTATTCCGTTAAAATATGTGATCTGCATCACA

4959 P3P6F TGGTTTGTTGGTAAGTATTCCGTGTTGGTATGTGATCTGCATCACA

5193 P4 AAGAATTTGGTGTTGACGTACCCCTATTCAGCAGTGTGGAACAGAAAAAGTGAACATTAG

5444 P4.1 AAGAATTTGGTGTTGACGTACCCCTATTCAGCAGTGTGGCATAGAAAAAGTGAACATTAG

5191 P5.1 TAAATCAAATGAGCTTATTTTTAACAGCGGAGGGCGTCTGCCGTGACGAGATTAATTAAT

5192 P5.2 TAAATCAAATGAGCTTATTTTTAACAGCGGAGGGCGTCTGCTGTGACGAGATTAATTAAT

5443 P5.3 TAAATCAAATGAGCTTATTTTTAACAGCGGAGGGCGTACGCTGTGACGAGATTAATTAAT

5473 P6.1F GTATTCCGTTCAAACATGTGATCTGCATCACAT

5474 P6.1R AGATCACATGTTTGAACGGAATACTTACGATAAAAC

5471 P6.2F GTATTCCGTTCAAATATGTGATCTGCATCACAT

5472 P6.2R AGATCACATATTTGAACGGAATACTTACGATAAAAC

4963 P6R CCAACTGTGATGCAGATCACATACCAACACGGAATACTT

4962 P6F AAGTATTCCGTGTTGGTATGTGATCTGCATCACAGTTGG

5196 P6.1 AAAGCCATGCTGATGGTTTTATCGTAAGTATTCCGTTCAAACATGTGATCTGCATCACAT

5197 P6.2 AAAGCCATGCTGATGGTTTTATCGTAAGTATTCCGTTCAAATATGTGATCTGCATCACAT
