# Supplementary material for: The Effect of Cell Growth Phase on the Regulatory Cross-Talk between Flagellar and Spi1 Virulence Gene Expression
Source: PLoS Pathog. 2014 Mar 6;10(3):e1003987. doi: 10.1371/journal.ppat.1003987 (PMC3946378; doi:10.1371/journal.ppat.1003987)
Supplement: Text S1 — Analysis of the promoters of the flhDC operon. (DOCX) [file ppat.1003987.s008.docx]

**Text S1**

**Analysis of the promoters of the *flhDC* operon**

Six transcriptional start sites (TSSs) have been identified in the regulatory region of the *Salmonella* *flhDC* operon. These TSSs were identified by primer extension (P1, P2, P3, P4, P5 and P6) [[1](#_ENREF_1)]. Using RNA-Seq based methods [[2](#_ENREF_2)] only P1, P3, P4 and P5 were identified. Each of the TSSs was preceded by a -10 box harboring A2 and T6, known conserved residues, except for P4 (Figure S3A). We have identified the P1 and P5 as bona fide functional promoters in a wild-type strain grown in LB at 30°C. To analyze the authenticity of the TSSs, P2, P3, P4 and P6 we generated variants by changing to a cytosine residue the conserved nucleotides A2 and T6 (Figure S3A) of the -10 of these TSSs in the duplicated region of the *flhDC* promoter driving transcription of the luciferase operon. These strains also harbor a wild-type copy of *flhDC* regulatory region driving the transcription of the *flhDC* operon. We demonstrated that mutations of nucleotides A2 or T6 in P2*_flhDC_* (P2^-^) or mutations of nucleotides that are not supposed to significantly alter the RNA-polymerase recognition of the -10 sequence reduced transcription of *flhDC* (Figure S3B). Mutations in P6*_flhDC_* (P6^-^) also reduced transcription of *flhDC* (Figure S3E). The -10 of P1*_flhDC_* and P6*_flhDC_* overlap with the binding site of CRP (Figure 4A), which is an activator of P1*_flhDC_* promoter transcription [[3](#_ENREF_3)]. We observed that a *crp* null mutation did not affect significantly the expression of *flhDC* in P6^-^.1 *_flhDC_* and P2^-^.1 *_flhDC_* mutants strains (Figure S3G) suggesting that mutations in the P2*_flhDC_* and P6*_flhDC_* likely affected transcription of the *flhDC* operon from the CRP-regulated P1*_flhDC_* promoter [[1](#_ENREF_1),[3](#_ENREF_3)]. Mutations in P3, the crucial and non-crucial residues, either did not change, increased or decreased transcription of *flhDC* (Figure S3C). Because P3*_flhDC_* promoter overlaps with the LrhA binding site (Figure 4A), the nucleotides substitutions made to study P3*_flhDC_* might have affected the LrhA binding to its DNA target at the *flhDC* regulatory region. In addition all mutations in P4*_flhDC_*, resulted in a significant increase of the *flhDC* transcription (Figure S3D).

The transcription of *flhDC* operon in P1^-^*_flhDC_* strain (P5-expressed) recapitulated what was observed in a P5^+^*_flhDC_* (Only P5 is functional while the other promoters are mutated). However, transcription of *flhDC* in the P5^-^*_flhDC_* (P1-expressed) construct was different from the transcription of *flhDC* in the P1^+^*_flhDC_* (Only P1 is functional while the rest of the promoters are mutated) construct. There was low level of expression in the P1^+^ compared to the level of expression of P5^-^ (P1-expressed). This difference is due to the fact that P1^+^*_flhDC_* harbors mutations P2^-^*_flhDC_* and P6^-^*_flhDC_*, which affect CRP-dependent transcription from P1. To test this hypothesis, we engineered two additional strains P6^+^2^+^1^+^*_flhDC_* and P5^+^6^+^2^+^ *_flhDC_* (Figure S3F) and demonstrated that these two strains recapitulated the transcription activities of strains P5^-^*_flhDC_* and P1^-^*_flhDC_*, respectively.

References

1. Yanagihara S, Iyoda S, Ohnishi K, Iino T, Kutsukake K (1999) Structure and transcriptional control of the flagellar master operon of Salmonella typhimurium. Genes Genet Syst 74: 105-111.

2. Kroger C, Dillon SC, Cameron AD, Papenfort K, Sivasankaran SK, et al. (2012) The transcriptional landscape and small RNAs of Salmonella enterica serovar Typhimurium. Proc Natl Acad Sci U S A 109: E1277-1286.

3. Soutourina O, Kolb A, Krin E, Laurent-Winter C, Rimsky S, et al. (1999) Multiple control of flagellum biosynthesis in Escherichia coli: role of H-NS protein and the cyclic AMP-catabolite activator protein complex in transcription of the flhDC master operon. J Bacteriol 181: 7500-7508.
